# Herpesvirus deconjugases inhibit the IFN response by promoting TRIM25 autoubiquitination and functional inactivation of the RIG-I signalosome

**DOI:** 10.1371/journal.ppat.1006852

**Published:** 2018-01-22

**Authors:** Soham Gupta, Päivi Ylä-Anttila, Simone Callegari, Ming-Han Tsai, Henri-Jacques Delecluse, Maria G. Masucci

**Affiliations:** 1 Department of Cell and Molecular Biology, Karolinska Institutet, Stockholm, Sweden; 2 Deutsches Krebsforschungszentrum, Heidelberg, Germany; University of Southern California, UNITED STATES

## Abstract

The N-terminal domains of the herpesvirus large tegument proteins encode a conserved cysteine protease with ubiquitin- and NEDD8-specific deconjugase activity. The proteins are expressed during the productive virus cycle and are incorporated into infectious virus particles, being delivered to the target cells upon primary infection. Members of this viral enzyme family were shown to regulate different aspects of the virus life cycle and the innate anti-viral response. However, only few substrates have been identified and the mechanisms of these effects remain largely unknown. In order to gain insights on the substrates and signaling pathways targeted by the viral enzymes, we have used co-immunoprecipitation and mass spectrometry to identify cellular proteins that interact with the Epstein-Barr virus encoded homologue BPLF1. Several members of the 14-3-3-family of scaffold proteins were found amongst the top hits of the BPLF1 interactome, suggesting that, through this interaction, BPLF1 may regulate a variety of cellular signaling pathways. Analysis of the shared protein-interaction network revealed that BPLF1 promotes the assembly of a tri-molecular complex including, in addition to 14-3-3, the ubiquitin ligase TRIM25 that participates in the innate immune response via ubiquitination of cytosolic pattern recognition receptor, RIG-I. The involvement of BPLF1 in the regulation of this signaling pathway was confirmed by inhibition of the type-I IFN responses in cells transfected with a catalytically active BPLF1 N-terminal domain or expressing the endogenous protein upon reactivation of the productive virus cycle. We found that the active viral enzyme promotes the dimerization and autoubiquitination of TRIM25. Upon triggering of the IFN response, RIG-I is recruited to the complex but ubiquitination is severely impaired, which functionally inactivates the RIG-I signalosome. The capacity to bind to and functionally inactivate the RIG-I signalosome is shared by the homologues encoded by other human herpesviruses.

## Author summary

The N-terminal domains of the herpesvirus large tegument proteins encode a conserved cysteine protease with ubiquitin- and NEDD8-specific deconjugase activity that was shown to regulate different aspects of the virus life cycle including virus replication, the assembly of infectious virus particles and the host innate anti-viral response. However, only few substrates have been identified, and the mechanisms of these effects remain largely unknown. We have used co-immunoprecipitation and mass spectrometry to identify cellular proteins that interact with Epstein-Barr virus (EBV) encoded homologue BPLF1. Several members of the 14-3-3 family of scaffold proteins were found amongst the top hits of the BPLF1 interactome. Analysis of the shared protein-interaction network revealed that BPLF1 promotes the assembly of a tri-molecular complex that includes 14-3-3 and the ubiquitin ligase TRIM25. The 14-3-3:TRIM25 complex participates in the innate immune response via Lys-63-linked ubiquitination of cytosolic pattern recognition receptor RIG-I. The capacity of BPLF1 to regulate this signaling pathway was confirmed by inhibition of the type-I IFN in cells transfected with a catalytically active BPLF1 N-terminal domain or expressing the endogenous protein upon reactivation of the productive virus cycle. We found that BPLF1 promotes the dimerization and autoubiquitination of TRIM25. RIG-I is recruited to the complex upon triggering of the IFN response but ubiquitination is severely impaired, which functionally inactivated the RIG-I signalosome. The capacity to promote TRIM25 autoubiquitination and inhibit the ubiquitination of RIG-I is shared by the homologues encoded by other human herpesviruses, providing a new insight on the mechanism by which these viruses halt the host innate immune antiviral response.

## Introduction

Virus infection is accompanied by extensive cellular changes caused by the pathogen to promote replication and spread, and by the host cell to fight the invasion. Viral gene products initiate these changes by interfering with a variety of cellular signaling pathways and gene expression programs. Post-translational modifications by covalent attachment of ubiquitin (Ub) and ubiquitin-like (UbL) polypeptides, such as SUMO, NEDD8 or ISG15, control a multitude of cellular functions by regulating protein turnover, localization, interactions and enzymatic activities [1]. The conjugation of Ub and UbLs to their substrates is mediated by a cysteine-based enzymatic cascade that comprises an ATP-dependent activating enzyme (E1), a conjugating enzyme (E2) and a substrate-specific ligase (E3) that transfers the modifier to the acceptor protein [2]. The modification is reversed by deconjugases that hydrolyze the covalent bond formed between the modifier and the substrate [3].

Components of the Ub and UbL modification machineries are targeted by viruses during different phases of the infection to promote various aspects of the virus life cycle and to inhibit intrinsic cellular defenses and the activation of innate and adaptive immune responses [4]. Two major viral strategies for interference with Ub and UbL signaling have been characterized. On the one side, viral proteins can hijack cellular components of the Ub and UbL conjugation machineries to redirect their activity towards new substrates whose modification favors infection. The first reported example of this strategy is the E6 protein of oncogenic human papilloma viruses (HPV) that retargets the cellular ubiquitin ligase E6AP to promote the degradation of the p53, which is required for expanding the pool of virus infected cells [5]. Several viral proteins were shown to function as substrate adaptors for Cullin-RING-Ligases (CRLs), extending the substrate range of the cellular enzymes [6, 7]. Deconjugases, such as the ubiquitin specific protease USP7-HAUSP, are similarly targeted by herpes simplex virus (HSV) ICP0 and Epstein-Barr virus (EBV) nuclear antigen (EBNA)-1 to alter the turnover of cellular proteins that regulate cell proliferation and apoptosis [8]. In addition, viruses may encode functional homologs of ligases and deconjugases that often share little sequence homology with their cellular counterparts and are therefore attractive targets for selective inhibition.

An interesting example of virus encoded functional homologs of cellular enzymes is the conserved family of herpesvirus deconjugases. The N-terminal domains of the major tegument proteins of herpesviruses show very little sequence similarity, but all the homologs investigated to date contain a conserved cysteine protease with potent Ub and NEDD8 specific deconjugase activity [9, 10]. The proteins are expressed during the early and late phases of the productive virus cycle and are incorporated into infectious virus particles [11, 12], suggesting that the enzyme may have multiple functions during the early and late phases of infection. Studies performed with recombinant viruses carrying mutations that ablate the protein or selectively inactivate the enzymatic activity have suggested important roles in the regulation of the virus production [13], infectivity [14, 15], and antiviral innate and adaptive immunity, including inhibition of NF-κB signaling and type I IFN responses [16–20]. However, the precise mechanisms of these effects and the primary substrates of the viral enzymes remain largely unknown. This is mostly due to the huge size of the intact viral protein, which hampers functional studies based on ectopic expression. This technical obstacle has been partly overcome in biochemical assays by using shorter constructs expressing only the N-terminal catalytic domain that is detected as a processing product in cells infected with some of the herpesviruses [9, 21]. Using this approach several putative substrates have been identified, including regulators of the cell cycle, components of the DNA damage response and repair pathways and proteins involved in the regulation of immune responses.

In this study, we have taken an unbiased approach based on co-immunoprecipitation and mass spectrometry to identify interacting partners and substrates of BPLF1, the viral deconjugase encoded by Epstein-Barr virus (EBV) a gamma-herpesvirus that establishes lifelong asymptomatic infections in the majority of adults worldwide [22]. Although largely harmless, the virus is also causally associated with a benign lymphoproliferative disease, infectious mononucleosis (IM), and participates in the pathogenesis of a broad spectrum of malignancies of lymphoid and epithelial origin [23]. Previous studies have implicated BPLF1 in the regulation of EBV DNA replication, the cell cycle, DNA damage and the host immune responses via interaction with a variety of putative viral [24] and cellular substrates including cullin-based E3 ligases [10], the DNA polymerase processivity factor PCNA [25], and components of the NF-κB signaling pathway such as TRAF6 [16, 17]. Analysis of the BPLF1 interactome identified the 14-3-3 family of scaffold proteins as major interacting partners. Search for E3 ligases that interact with both 14-3-3 and BPLF1 pointed to the IFN-β signaling as a likely target. In line with this possibility, we found that expression of a catalytically active BPLF1 is associated with impaired IFNβ response in transfected cells, and under physiological conditions of expression in EBV positive AGS-Bx1 and HEK293-EBV cells that carry recombinant replication competent EBV strain. Biochemical analysis revealed that BPLF1 forms a tri-molecular complex with 14-3-3 and the TRIM25 ubiquitin ligase. The formation of the complex correlates with TRIM25 autoubiquitination and is associated with failure to ubiquitinate RIG-I and consequent inactivation of the downstream signaling events.

## Results

### The 14-3-3 scaffold proteins are interacting partners of BPLF1

**Fig 1:**
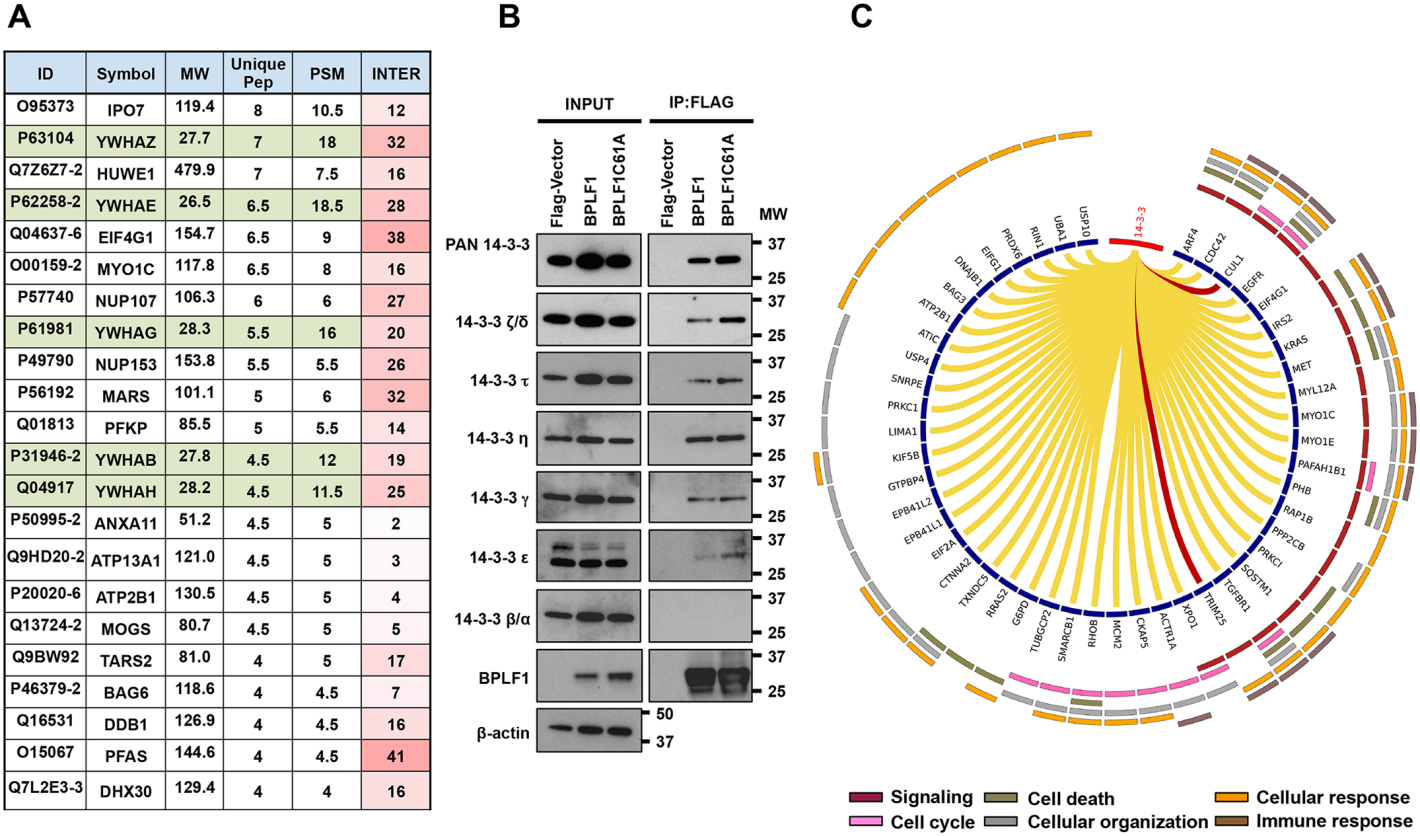
BPLF1 interacting proteins identified by tandem mass spectrometry. **A**. List of the 22 most abundant BPLF1 interacting proteins identified by mass spectrometry. The values are derived from the mean of two independent experiments. ID = protein ID, MW = molecular weight, Unique Pep = number of unique peptides mapping to the protein, PSM = number of peptide spectrums matched to the protein. INTER = number of annotated interacting proteins found in the BPLF1 interactome. 14-3-3 isoforms: YWHAZ = 14-3-3 ζ/δ, YWHAE = 14-3-3 ε, YWHAG = 14-3-3 γ, YWHAB = 14-3-3 β/α, YWHAH = 14-3-3 η. **B**. The interaction of BPLF1 with the 14-3-3 isoforms was validated by immunoprecipitation and immunoblotting with the indicated isoform-specific antibodies. **C**. Circos diagram illustrating the annotated interaction of the 14-3-3 isoforms with other proteins enriched in the BPLF1 interactome. The two ubiquitin ligases found in the shared interactome, CUL1 and TRIM25, are highlighted in red. Color coded annotated functions are shown in the outer ring.

In order to achieve an unbiased overview of the BPLF1 interactome and to identify putative substrates, FLAG-tagged versions of the catalytically active N-terminal domain of BPLF1 (FLAG-BPLF1 aa 1–325), or a catalytic mutant where the Cys61 residue was mutated to Ala (FLAG-BPLF1C61A) were transfected in HeLa cells along with a FLAG-empty vector control, and interacting proteins were immunoprecipitated using anti-FLAG conjugated agarose beads. After elution with the FLAG peptide, the immunoprecipitates were fractionated by SDS-PAGE and slices of gels obtained from two independent experiments were subjected to tandem mass spectrometry, MS/MS (Supporting Information, S1A Fig). Two hundred and seventy-seven polypeptides identified by at least two unique spectral counts were detected in both immunoprecipitates from cells expressing FLAG-BPLF1, but not the FLAG-empty vector control. Of these, 72 were also detected in one or both immunoprecipitates of cells expressing FLAG-BPLF1C61A, while 18 polypeptides were detected only in immunoprecipitates of the catalytic mutant BPLF1 (Supporting Information, S1B Fig). Gene Ontology analysis revealed that the BPLF1 interactome is enriched in proteins involved in transcription and RNA metabolism, nuclear transport and protein trafficking, cell cycle and apoptosis, and ubiquitin dependent cellular processes (Supporting Information, S1C Fig). Analysis of the protein interaction network identified a major interacting hub centering around several proteasome subunits and additional hubs involving the EGFR, nuclear transport proteins and several members of the 14-3-3 family of adaptor proteins (Supporting Information, S1D Fig). A list of 22 hits that bound exclusively to catalytically active and inactive BPLF1 and were identified by 4 or more unique peptides is shown in Fig 1A. Approximately one fourth of these top hits were members of the 14-3-3- family, including 14-3-3ζ, -ε, -γ, -β and -η (highlighted in green in Fig 1A). A proteome network analysis revealed that the 14-3-3 proteins also share a high number of interacting partners with BPLF1 (Fig 1A, column 6) suggesting that they may be found in protein complexes that regulate different cellular functions.

To gain insight on the functional significance of the interaction of BPLF1 with 14-3-3 proteins we first sought to validate the interaction in co-immunoprecipitation assays. Lysates of HeLa cells transfected with FLAG-BPLF1(aa 1-235), FLAG-BPLF1C61A or FLAG-empty vector were immunoprecipitated with anti-FLAG conjugated agarose beads and western blots of the FLAG peptide eluted proteins were probed with a pan-14-3-3 specific antibody or antibodies specific for different 14-3-3 isoforms. A representative western blot where all the antibodies were tested in parallel is shown in Fig 1B. All 14-3-3 isoforms identified by MS/MS were also detected in western blots probed with isoform-specific antibodies except for 14-3-3β. Minor differences in the efficiency of co-immunoprecipitation of the 14-3-3 isoforms with wild type and mutant BPLF1 were not reproduced in repeated experiments, confirming that the interaction is not dependent on enzymatic activity. Of note, bioinformatics analysis failed to identify a canonical 14-3-3 binding site in BPLF1 and the efficiency of co-immunoprecipitation was not affected by phosphatase treatment of the lysates (Supporting Information, S2 Fig), suggesting that the phosphorylation-dependent binding motif of 14-3-3 is not involved in the interaction.

Although the abundance of several 14-3-3 isoforms is regulated by ubiquitination and proteasome-dependent degradation [26, 27], we could not demonstrate any significant effect of BPLF1 on the steady state of the different 14-3-3 isoforms (Supporting Information, S3A Fig). Thus, 14-3-3 proteins may not serve as physiologic substrates of the BPLF1 deconjugase, even though deubiquitination may occur when the modification is forced by overexpression of HA-tagged ubiquitin (Supporting Information, S3B Fig). We turned therefore to the analysis of annotated 14-3-3 interacting partners that are also found in the BPLF1 interactome with the aim to identify cellular functions and signaling pathways that may be regulated by the two proteins. As illustrated by the Circos diagram shown in Fig 1C, 48 proteins that are predicted to interact with both 14-3-3 and BPLF1 participate in a variety of cellular functions including signaling, the cell cycle, apoptosis, cellular organization and immune responses. The shared interactome included two E3 ligases, CUL1 and TRIM25 (highlighted by red arrows in Fig 1C) that may be part of functional complexes whose activity is regulated by BPLF1. We have previously shown that the interaction of BPLF1 with Cullin-based ubiquitin ligases regulates their function via deNEDDylation of the cullin scaffold [10]. The TRIM25 ligase interacts with several 14-3-3 isoforms and, together with 14-3-3ε was shown to regulate the RIG-I signalosome that mediates the activation of type I IFN in virus infected cells [28], suggesting that BPLF1 may interfere with the activity of this signaling complex.

### BPLF1 inhibits the type I interferon (IFN) response

**Fig 2:**
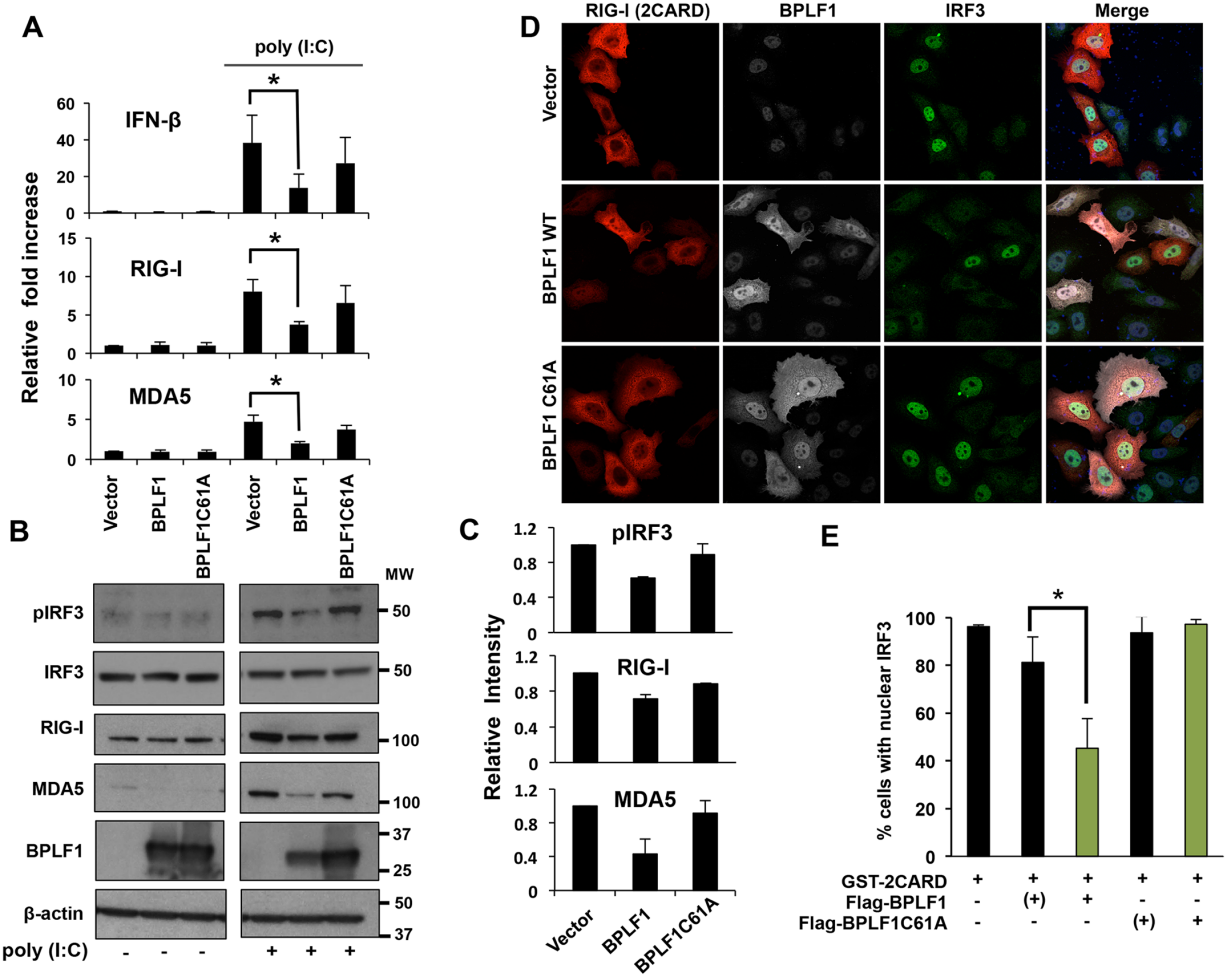
Catalytically active BPLF1 inhibits the IFN response. **A**. BPLF1 inhibits the transcription of IFN and IFN-regulated genes induced by treatment with poly(I:C) for 20 hrs. The levels of specific mRNAs were quantified by qRT-PCR. The results are shown as fold change relative to mock treated cells. The mean ± SD of 3 experiments each performed in triplicate is shown. **B**. The phosphorylation of IRF3 and expression of the nucleic acid sensors RIG-I and MDA5 are downregulated in cells expressing active BPLF1. Representative western blots illustrating the protein expression levels of the samples shown in Fig. 2A probed with the indicated specific antibodies. **C**. The intensities of the specific bands were quantified by densitometry and fold change was calculated relative to the empty vector transfected cells. The mean ± SD of two experiments is shown. **D**. Active BPLF1 inhibits the nuclear translocation of IRF3 in cells activated by transfection with GST-2CARD. Confocal images were obtained at 63x lens objective magnification. IRF3 is in green, RIG-I-2CARD is in red, BPLF1 in far red and cell nuclei were stained with Hoechst (blue). **E**. Statistical analysis of the percentage of cells exhibiting nuclear IRF3. The bars depict the average percentage of cells with nuclear IRF3 in cells expressing GST-2CARD with or without BPLF1 co-expression. BPLF1 transfected cells that did not express detectable amounts of the protein are indicated as (+). The mean ± SD of three independent experiments is shown.

The 14-3-3 scaffold proteins participate in the IFN response by interacting with TRIM25 and the nucleic acid sensors RIG-I [28], which promotes K63-linked ubiquitination and is essential for translocation to Mitochondrial Anti-Viral Signaling (MAVS) adaptors [29]. Subsequent ubiquitin-regulated signaling events lead to phosphorylation and nuclear translocation of IRF3 and transcriptional activation of IFN and IFN-regulated genes, including RIG-I and MDA5 [30]. In order to investigate whether BPLF1 regulates this arm of the innate antiviral response, the transcription of IFNβ, RIG-I and MDA5 was induced by treatment with poly(I:C) in HeLa cells transfected with FLAG-BPLF1, FLAG-BPLF1C61A or the control FLAG-empty vector, and the levels of the specific mRNA were quantified by qPCR. As expected, treatment with poly(I:C) was associated with a strong induction of the three transcripts in control cells (Fig 2A). Significantly reduced levels of the specific mRNAs were observed in cells expressing the catalytically active BPLF1, whereas expression of the BPLF1C61A mutant had no appreciable effect (Fig 2A), suggesting that the enzymatic activity of BPLF1 is required for inhibition of the IFN response. This effect was confirmed by a reproducible decrease of RIG-I and MDA5 protein levels detected in western blots probed with the specific antibodies (Fig 2B and 2C). In addition, the levels of phosphorylated IRF3 were also decreased by approximately 50% (Fig 2B and 2C), suggesting that BPLF1 acts upstream of this signaling event. To confirm this finding, we took advantage of the notion that, after phosphorylation in the cytoplasm, pIRF3 is translocated to the nucleus where the activated transcription factor binds to the promoter of IFN responsive genes [31]. The number of cells exhibiting cytoplasmic or nuclear IRF3 fluorescence was scored in confocal images of control cells and cells expressing the catalytically active and mutant BPLF1 stained with an IRF3-specific antibody. Since treatment with poly(I:C) was associated with poor survival of cells expressing active BPLF1, the IFN response was activated by transfection of a constitutively active RIG-I construct (GST-2CARD) [32], which also allowed direct visualization of the activated cells. Expression of the catalytically active BPLF1 impaired the nuclear translocation of IRF3, with IRF3 fluorescence being virtually undetectable in the nuclei of most BPLF1 positive cells compared to BPLF1 negative cells present in the same transfected sample (Fig 2D and 2E). Expression of BPLF1C61A had no appreciable effect, confirming that enzymatically active BPLF1 is required for this effect.

**Fig 3:**
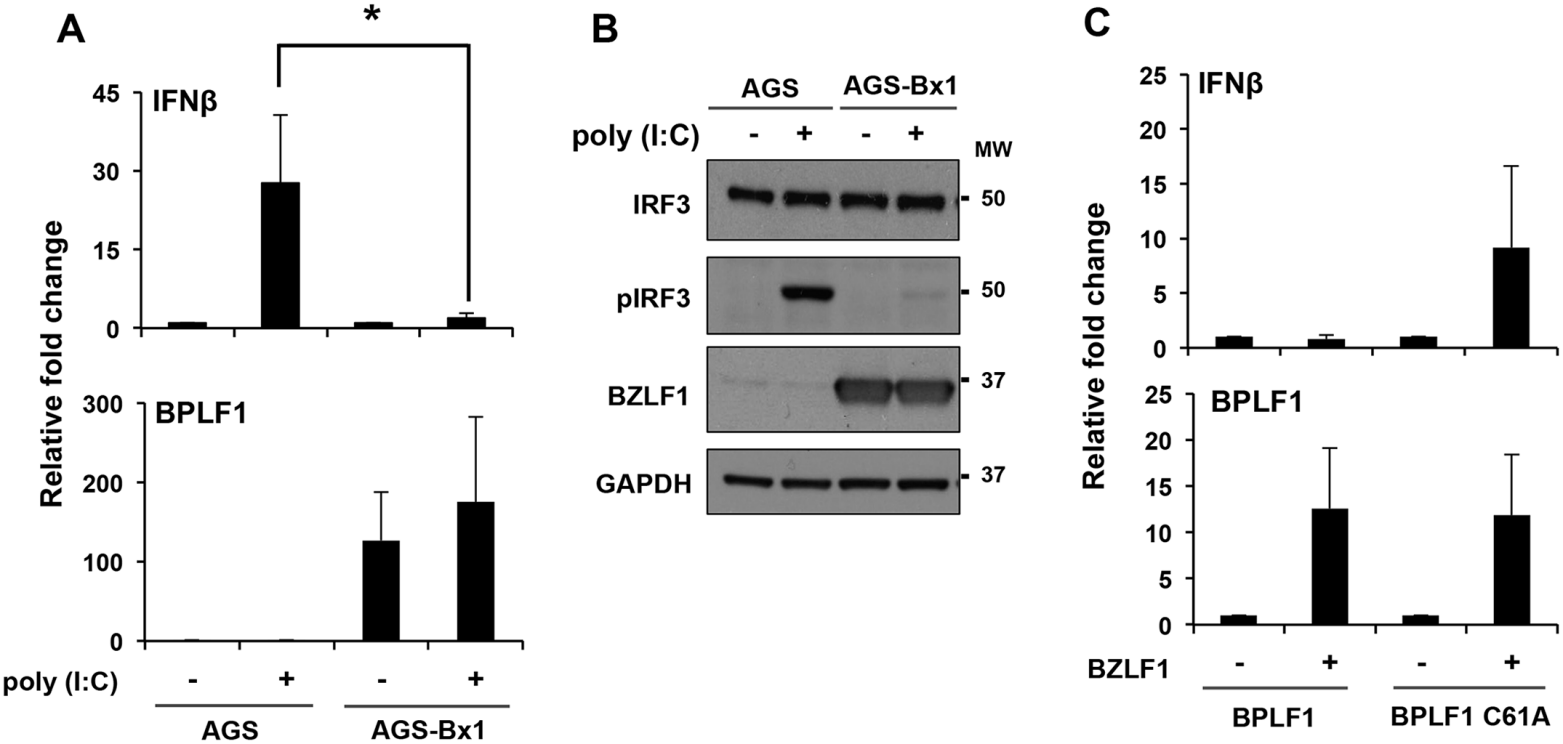
Endogenous BPLF1 inhibits the IFN response upon reactivation of the productive virus cycle. **A**. EBV negative AGS and the EBV converted AGS-Bx1 cell lines were treated for 48 hrs with TPA/Bu and the IFN response was activated by transfecting with 2 μg/ml poly(I:C) for 6 hrs. The expression of IFNβ and BPLF1 mRNA was quantified by qRT-PCR. The mean ± SD of two experiments is shown. **B**. Western blots of cell lysates were probed with the indicated antibodies. One representative experiment out of 3 is shown. **C**. HEK293 cells carrying recombinant EBV strains encoding for WT or C61A mutated BPLF1 were transfected with a BZLF1 plasmid for induction of the productive virus cycle. The transcript levels of IFNβ and BPLF1 were quantified by qRT-PCR, using GAPDH as a reference gene. The mean ± SD of 3 independent experiments is shown.

In order to investigate whether BPLF1 may exhibit this inhibitory activity also under physiological conditions of expression, we took advantage of two sets of cell pairs including EBV negative AGS and EBV converted subline AGS-Bx1, and HEK293 cells that carry recombinant EBV strains encoding for wild type or catalytic mutant BPLF1. The IFN pathway was triggered by treatment with poly(I:C) in AGS and AGS-Bx1 cells exposed to TPA/Bu for 48 hrs and the efficiency of the response was assessed by monitoring IFNβ mRNA and IRF3 phosphorylation. Expression of BPLF1 correlated with significantly reduced levels of IFNβ mRNA in TPA/Bu treated AGS-Bx1 compared to TPA/Bu treated EBV negative AGS (Fig 3A). IRF3 phosphorylation was virtually undetectable in the EBV positive cells (Fig 3B), confirming that an early event in the signaling cascade is affected. The possibility that endogenously expressed BPLF1 may be required for this inhibitory effect was substantiated by comparing the expression of IFNβ mRNA in induced EBV positive HEK293 cells. As expected, induction of the productive virus cycle by transfection of the viral transactivator BZLF1 was associated with comparable upregulation of the BPLF1 mRNA in cells expressing the active or catalytic inactive versions of the enzyme (Fig 3C). However, while in cells carrying the active enzyme the levels of IFNβ mRNA remained low or even decreased relative to un-induced cells, a significant increase was observed in cells expressing BPLF1C61A, confirming the capacity of endogenously expressed BPLF1 to inhibit the IFN response.

### BPLF1 forms a tri-molecular complex with TRIM25 and 14-3-3 and promotes TRIM25 autoubiquitination

**Fig 4:**
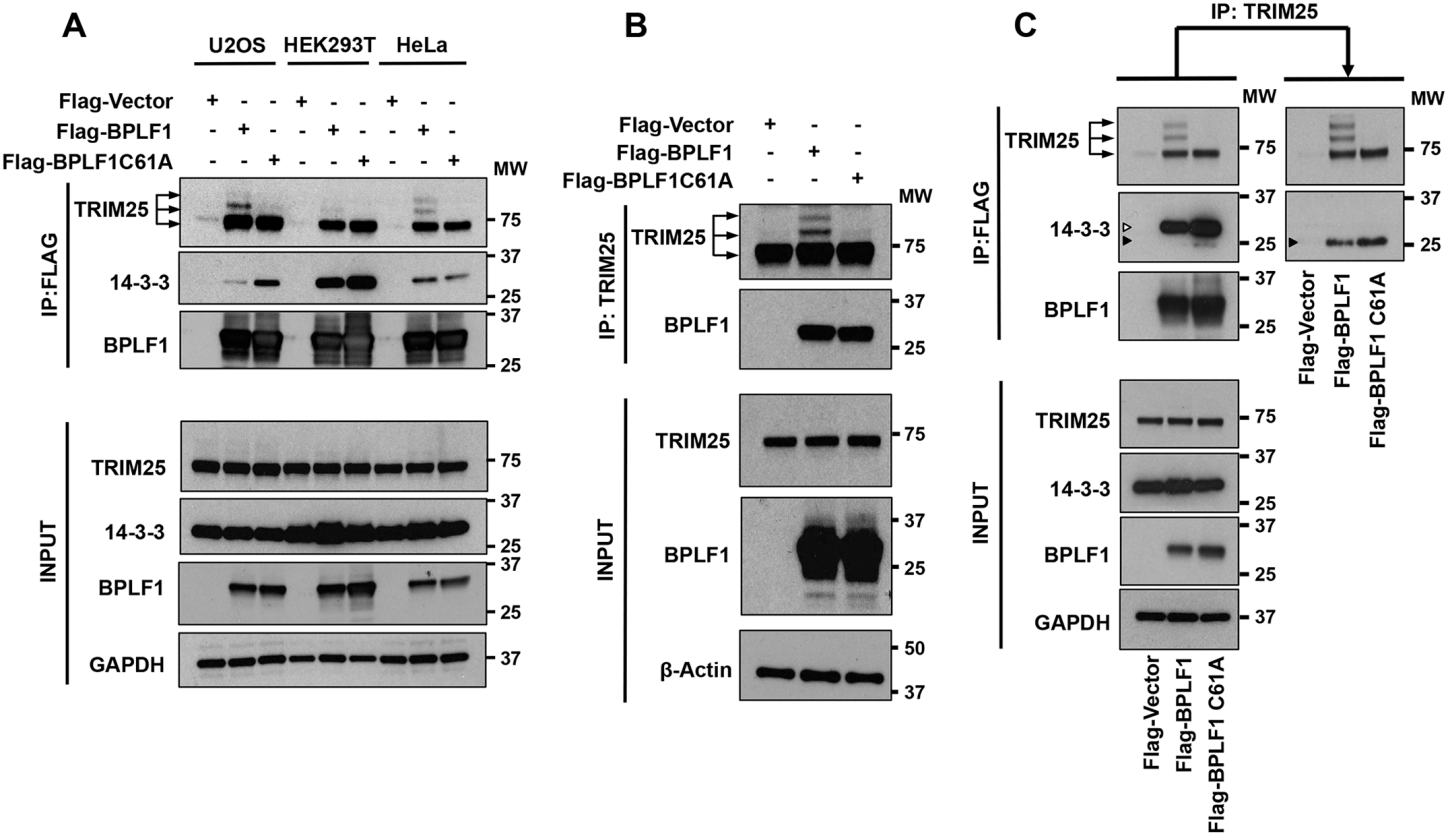
BPLF1 forms a tri-molecular complex with 14-3-3 and TRIM25. **A**. BPLF1 interacts with both 14-3-3 and TRIM25. HeLa, U2OS and HEK293T cells were transfected with the indicated plasmids and cell lysates were immunoprecipitated with anti-FLAG conjugated agarose beads (IP). Immunoblotting (IB) was performed with the indicated antibodies. **B**. TRIM25 interacts with BPLF1. HeLa cells were transfected with the indicated plasmids and cell lysates were immunoprecipitated with the anti-TRIM25 antibody (IP). Immunoblotting was performed with the indicated antibodies. **C**. BPLF1 forms a tri-molecular complex with 14-3-3 and TRIM25. Lysates of HeLa cells transfected with the indicated plasmids were immunoprecipitated with anti-FLAG conjugated beads and complexes eluted with the FLAG peptide were subjected to sequential immunoprecipitation with the anti-TRIM25 antibody. Immunoblots were probed with antibodies to 14-3-3 and TRIM25. The two 14-3-3 specific bands are indicated by unfilled (upper band) and filled arrows (lower band). One representative experiment out of 3 is shown.

Having observed that BPLF1 regulates an early step in the IFN signaling cascade, we then sought to characterize the mechanism of this effect. To this end, we first asked whether BPLF1, 14-3-3 and TRIM25 participate in the same signaling complex. Plasmids expressing FLAG-BPLF1, FLAG-BPLF1C61A and the FLAG-empty vector were transfected in U2OS, HEK293T and HeLa cells and FLAG-immunoprecipitates were probed with antibodies to 14-3-3 and TRIM25. Both 14-3-3 and TRIM25 were detected in the immunoprecipitates from the three cell lines confirming that the two proteins interact with BPLF1 (Fig 4A). In all cases, the interaction was independent of the activity of the viral enzyme, although, as discussed in later sections, a ladder of TRIM25 species was regularly detected in the immunoprecipitates of catalytically active BPLF1. The interaction of BPLF1 with TRIM25 was validated by reverse co-immunoprecipitations of endogenous TRIM25 (Fig 4B), but we were unable to immunoprecipitate endogenous 14-3-3 proteins using different pan- or isotype-specific antibodies. The 14-3-3 proteins were not detected in immunoprecipitates of endogenous TRIM25 performed under standard conditions, suggesting that only a small fraction of the endogenously expressed species may be part of the tri-molecular complex. To test this possibility, the endogenous TRIM25 recovered in FLAG-immunoprecipitates from cells expressing FLAG-BPLF1 and the catalytic mutant was subjected to a second round of immunoprecipitation using a TRIM25 specific antibody (Fig 4C). A 14-3-3 species that migrated slightly faster than the major species detected in the FLAG immunoprecipitates was strongly enriched in the complex. Since the different 14-3-3 isoforms appear to migrate with similar size (Fig 1B), the enriched species may carry different post translational modifications that regulate the binding properties of 14-3-3 [33]. Alternatively, the fast migrating species may also correspond to a previously described splice variant of 14-3-3ε that is expressed with low abundance in all tissues [34]. Collectively, these findings suggest that BPLF1 promotes the formation of a tri-molecular complex including 14-3-3 and the TRIM25 ubiquitin ligase.

**Fig 5:**
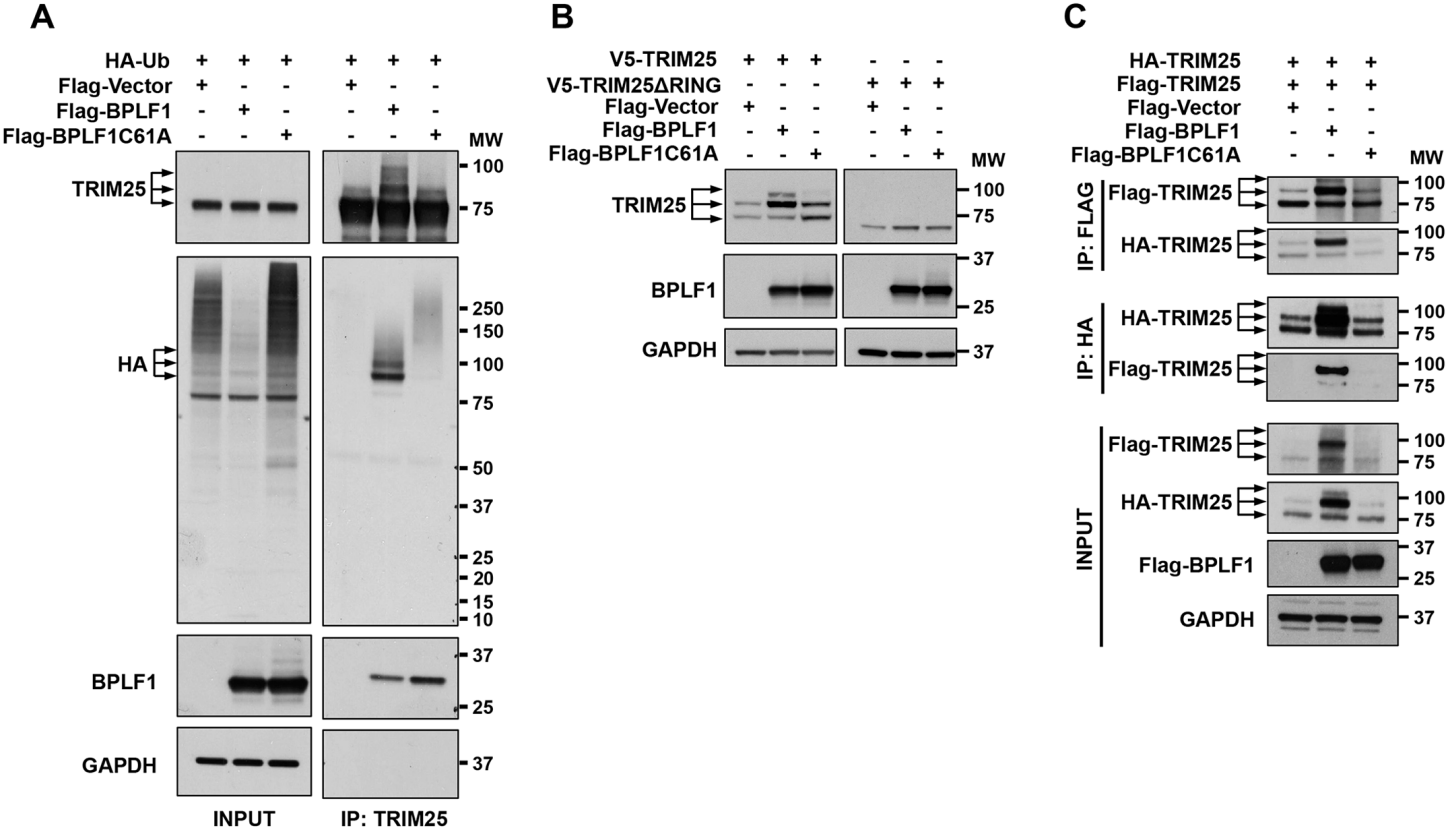
BPLF1 promotes TRIM25 dimerization and auto-ubiquitination. **A**. Catalytically active BPLF1 promotes TRIM25 ubiquitination. HA-Ub was co-transfected in HeLa cells together with the indicated FLAG-tagged plasmids. Cell lysates were immunoprecipitated with the anti-TRIM25 antibody, and immunoblotted with anti-HA antibodies. Unmodified, mono- and di-ubiquitinated TRIM25 species are indicated by arrows. One representative experiment out of 3 is shown. **B**. BPLF1 induces TRIM25 auto-ubiquitination. HeLa cells were transfected with either V5-TRIM25 or V5-TRIM25ΔRING together with the indicated FLAG-tagged plasmids. Total cell lysates were probed with an anti-V5 antibody. Unmodified, mono- and di-ubiquitinated TRIM25 species are indicated by arrows. One representative experiment out of 2 is shown. **C**. Active BPLF1 promotes the dimerization and functional activation of TRIM25. HeLa cells were co-transfected with the indicated FLAG-tagged plasmids and with HA- and FLAG-tagged TRIM25. Western blots of HA and FLAG immunoprecipitates were probed with antibodies to the HA and FLAG tags. One representative experiment out of 3 is shown.

The detection of a regularly spaced ladder of TIRM25 species of increasing size in the immunoprecipitates of catalytically active BPLF1 suggests that formation of the complex may promote modification of the ligase by conjugation with Ub or UbLs. Since TRIM25 is known to be modified by both Ub and ISG15 [35], FLAG-tagged BPLF1 and the catalytic mutant were co-transfected in HeLa cells together with HA-Ub or HIS-ISG15. Cell lysates were prepared in the presence of 1% SDS to resolve protein complexes and TRIM25 immunoprecipitates were probed with antibodies specific for the HA and HIS tags. Distinct bands of size corresponding to the high molecular weight species detected by the TRIM25-specific antibody were detected by the HA-antibody in cells expressing HA-Ub (Fig 5A), suggesting that BPLF1 promotes the ubiquitination of TRIM25. High molecular weight species were not detected in TRIM25 immunoprecipitated from cells expressing HIS-ISG15 (Supporting information, S4A Fig), indicating that TRIM25 is not ISGylated. It is noteworthy that, in line with the potent Ub deconjugase activity of BPLF1, the total level of ubiquitinated proteins was significantly lower in cells expressing the active enzyme compared to control cells and cells expressing the mutant BPLF1 (Fig 5A), suggesting that ubiquitinated TRIM25 is selectively protected from deubiquitination.

TRIM25 is a substrate of the LUBAC ubiquitin ligase [36] and may also undergo autoubiquitination [37], which is dependent on the RING domain. To test whether the interaction with BPLF1 promotes autoubiquitination, V5-tagged TRIM25 and a RING-deletion mutant (TRIM25ΔRING) were co-transfected in HeLa cells together with active or mutant BPLF1 and western blots were probed with a V5-specific antibody (Fig 5B). The V5 antibody detected the ectopically expressed TRIM25 as a doublet due to the appearance of a mono-ubiquitinated species that was removed when cysteine protease inhibitors were omitted from the lysis buffer (Supporting Information, S4B Fig). The intensity of the mono-ubiquitinated band was strongly increased in the presence of catalytically active BPLF1 and an additional di-ubiquitinated species was also detected (Fig 5B, left panel). In line with the possibility that binding to catalytically active BPLF1 may promote TRIM25 autoubiquitination, higher molecular weight species were not detected in cells expressing the TRIM25ΔRING mutant (Fig 5B, right panel).

The ligase activity of TRIM25 is dependent on dimerization and oligomerization, which bring the RING domains of four TRIM25 molecules in close proximity to the ubiquitin loaded E2 [37]. Thus, the appearance of auto-ubiquitinated species suggests that BPLF1 may promote the oligomerization of TRM25. To test this possibility, FLAG- and HA-tagged TRIM25 were co-expressed in HeLa cells together with catalytically active or inactive BPLF1, and western blots of complexes immunoprecipitated with FLAG- or HA-conjugated beads were probed with HA- and FLAG-specific antibodies. As illustrated by the representative western blot shown in Fig 5C, ubiquitinated forms of TRIM25 were reciprocally immunoprecipitated by HA- and FLAG-tagged TRIM25 in cells expressing the catalytically active BPLF1, confirming the formation of dimers and oligomers. It is noteworthy that a significant accumulation of the transfected TRIM25 was observed in cells expressing active BPLF1. This is likely to be an artifact of overexpression in the presence of the active DUB since BPLF1 did not significantly affect the turnover of the endogenous protein.

### BPLF1 promotes functional inactivation of the RIG-I signalosome

**Fig 6:**
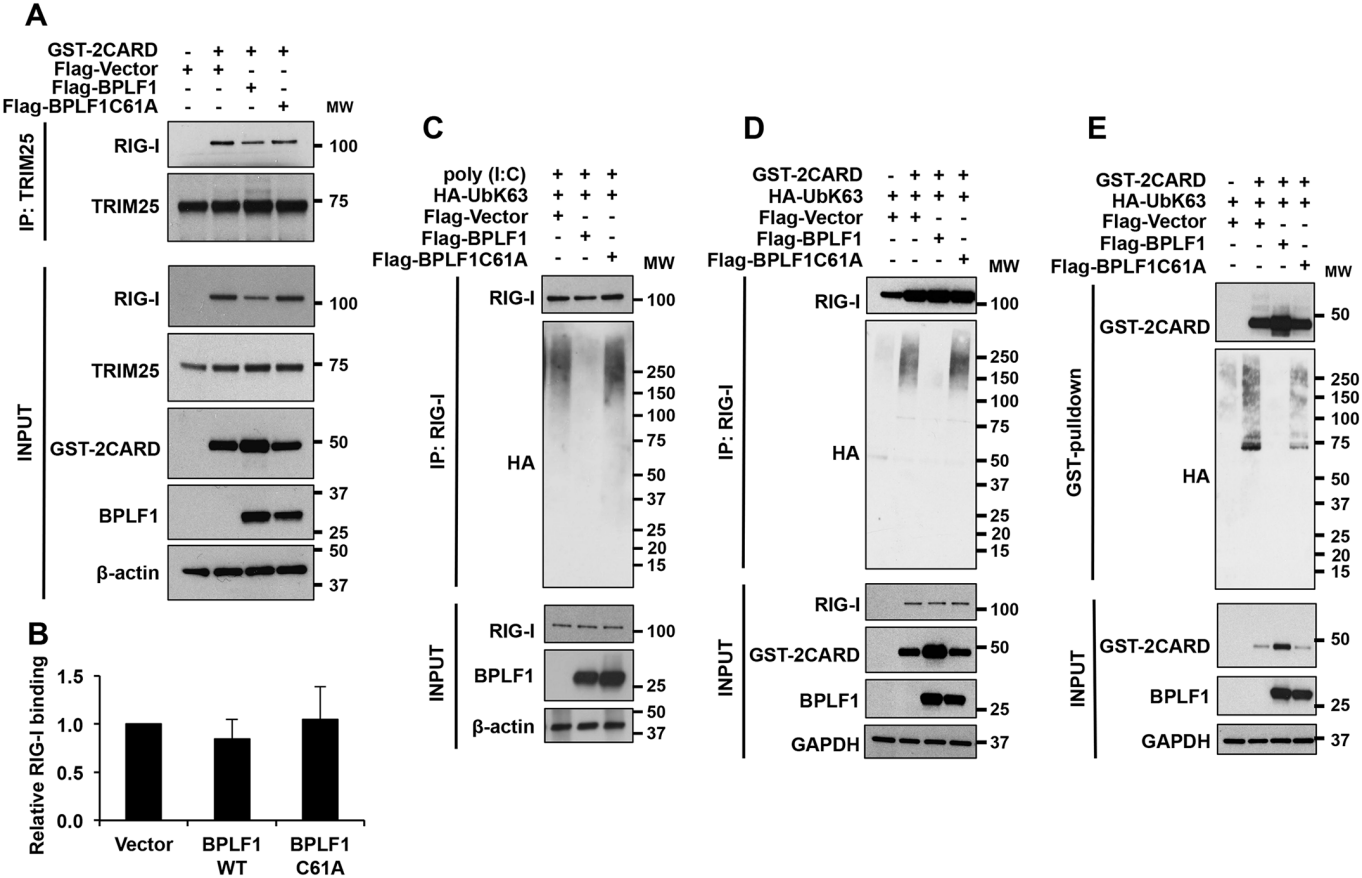
BPLF1 inhibits the ubiquitination of RIG-I. **A**. BPLF1 does not affect the interaction of TRIM25 with activated RIG-I. The IFNβ signaling pathway was induced in HeLa cells transfected with the indicated FLAG-tagged plasmid by co-transfection of the GST-2CARD. Endogenous TRIM25 was immunoprecipitated and western blots were probed with a RIG-I specific antibody. One representative experiment out of 3 is shown. **B**. The intensity of interaction of the RIG-I band was quantified by densitometry. The ratio of the intensity of the immunoprecipitated RIG-I band relative to the input is shown. The means ± SD of 3 experiments are shown. **C**. BPLF1 inhibits the ubiquitination of RIG-I in cells treated with poly(I:C). HA-UbK63 was co-transfected in HeLa cells together with the indicated FLAG-tagged plasmid. RIG-I ubiquitination was induced by transfecting the cells with 2 μg/ml poly(I:C) for 16 hrs. Endogenous RIG-I was immunoprecipitated and ubiquitination was detected by probing immunoblots with the anti-HA antibody. One representative experiment out of 2 is shown. **D**. BPLF1 inhibits the ubiquitination of endogenous RIG-I in cells expressing GST-2CARD. The experiment was performed as in Fig. 6C except that activation of the IFNβ pathway was induced by transfection of GST-2CARD. One representative experiment out of 2 is shown. **E**. BPLF1 inhibits the ubiquitination of GST-2CARD. GST-pulldown was performed from lysates of cells treated as in Fig 6D and western blots were probed with the anti-HA antibody. One representative experiment out of 2 is shown.

Having shown that BPLF1 promotes the formation of a 14-3-3:TRIM25 complex where the TRIM25 ligase is functionally activated, we then asked whether the complex still recruits and ubiquitinates activated RIG-I. To this end, the IFN response was triggered in cells expressing enzymatically active and inactive BPLF1 by co-transfection of the GST-2CARD construct. Activation of the pathway was associated with strong up-regulation of the endogenous RIG-I in vector-transfected cells, while, consistent with the inhibitory effect of BPLF1, a weaker band was detected in cells expressing the active enzyme (Fig 6A). Probing TRIM25 immunoprecipitates with a RIG-I specific antibody confirmed that RIG-I is recruited to the complex. Although minor differences were observed in repeated experiments, the recruitment of RIG-I was not significantly affected in cells expressing the active BPLF1 (Fig 6B). The efficiency of co-immunoprecipitation of the 2CARD polypeptide could not be reliably assessed using the anti-GST antibody because the specific band co-migrates with the heavy chain of the rabbit antibody used for TRIM25 immunoprecipitation.

TRIM25 activates the RIG-I signalosome by inducing K63-linked ubiquitination of RIG-I [38]. In order to test whether the recruitment of RIG-I to the TRIM25 complex leads to K63-linked ubiquitination, HeLa cells transfected with FLAG-tagged BPLF1, BPLF1C61A and control FLAG-empty vector were co-transfected with a plasmid expressing HA-UbK63 where all Lys residues in ubiquitin except K63 are mutated to Arg. Activation of the pathway by treatment with poly(I:C) (Fig 6C) or transfection of GST-2CARD (Fig 6D and 6E) was accompanied by the appearance of a smear of ubiquitinated RIG-I (Fig 6C and 6D) or GST-2CARD species (Fig 6E) in western blots of immunoprecipitates probed with the anti-HA antibody. The accumulation of ubiquitinated RIG-I was abrogated in the presence of active BPLF1, while expression of the BPLF1C61A mutant had no effect. Since the autoubiquitination of TRIM25 indicates that the ligase is active, the failure to accumulate ubiquitinated RIG-I suggest that the viral DUB recruited to the complex may directly counteract the activity of the ligase, which is in line with the capacity of BPLF1 to efficiently disassemble both K48- and K63-linked ubiquitin chains (Supporting Information, S4C Fig). Collectively, these findings support the conclusion that BPLF1 inhibits the IFN response by impairing the ubiquitination of RIG-I, preventing thereby the formation of an active RIG-I signalosome.

### The BPLF1 homologs share the capacity to regulate the RIG-I signalosome

**Fig 7:**
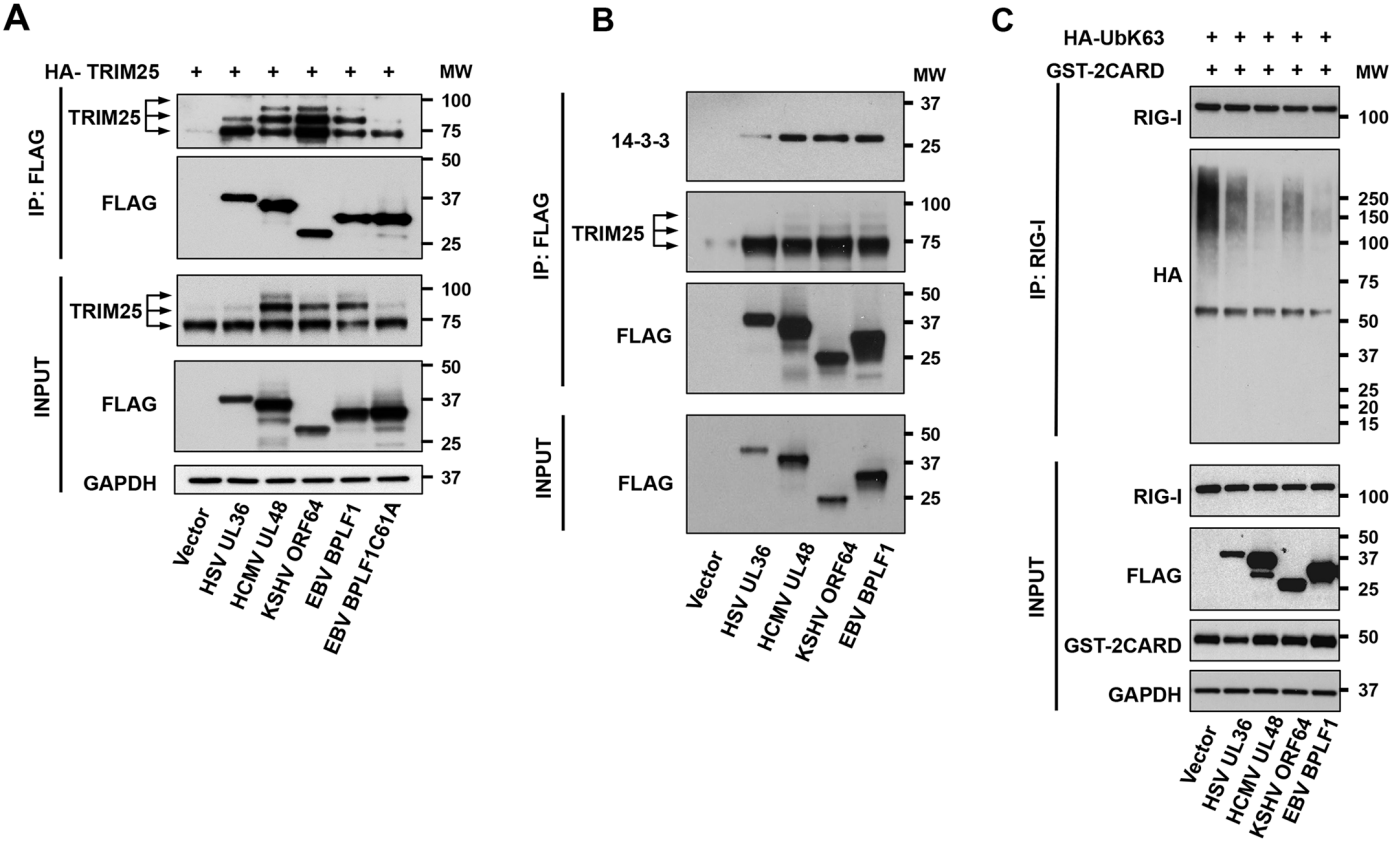
The effect of BPLF1 is conserved in homologs encoded by other herpesviruses. **A**. Functional homologs of BPLF1 encoded by HSV, HCMV and KHSV promote the autoubiquitination of TRIM25. HeLa cells were co-transfected with HA-TRIM25 plasmid and plasmids encoding the indicated catalytically active FLAG-tagged N-terminal domains of herpesvirus deconjugases along with catalytically inactive FLAG-BPLF1C61A. The viral proteins were immunoprecipitated with anti-FLAG-conjugated agarose beads and co-precipitated endogenous TRIM25 was detected in western blots probed with the specific antibody. Mono- and di-ubiquitinated TRIM25 species are indicated by arrows. One representative experiment is shown. **B**. The herpesvirus deconjugases interact with endogenous 14-3-3 and TRIM25. FLAG-immunoprecipitates were probed with the pan-14-3-3 and TRIM25 antibodies. **C**. The BPLF1 homologs inhibit the ubiquitination of RIG-I. HA-UbK63 was co-transfected in HeLa cells together with the indicated FLAG-tagged herpesvirus deconjugases. RIG-I ubiquitination was induced by co-transfecting the cells with GST-2CARD. Endogenous RIG-I was immunoprecipitated and ubiquitination was detected by probing immunoblots with the anti-HA antibody. One representative experiment out of 2 is shown.

In the final set of experiments, we asked whether the capacity of BPLF1 to interact with the RIG-I signalosome and inhibit its function is shared by the homologs encoded by other human herpesviruses. To this end, constructs expressing FLAG-tagged versions of the N-terminal catalytic domain of HSV-UL36, HCMV-UL48 and KHSV-ORF64 were transfected in HeLa cells along with BPLF1 and the mutant BPLF1C61A. The enzymatic activity of the viral proteins was confirmed by labelling cell lysates with the functional probe Ub-VME (Supporting Information, S5 Fig). To ensure robust detection, the cells were co-transfected with HA-tagged TRIM25. Cell lysates were immunoprecipitated with anti-FLAG agarose beads and western blots were probed with the TRIM25-specific antibody. As illustrated by the representative experiment shown in Fig 7A, bands corresponding to mono- and di-ubiquitinated TRIM25 were strongly enriched in immunoprecipitates from cells expressing the active viral enzymes but not from cells expressing the inactive BPLF1C61A, confirming that the homologs share the capacity to promote dimerization and functional activation of TRIM25. All the homologues co-precipitated both 14-3-3 and TRIM25 (Fig 7B), suggesting that they share the capacity to form a trimolecular complex with the cellular proteins. The relevance of the interaction for regulation of the RIG-I signalosome was further supported by the failure to detect K63-ubiquitinated RIG-I in cells expressing the active viral enzymes (Fig 7C).

## Discussion

In this study, we provide evidence for the capacity of the deconjugases encoded within the large tegument proteins of human herpesviruses to regulate the innate anti-viral response by binding to and promoting functional inactivation of the RIG-I signalosome. Interference with downstream events in this signaling cascade, such as the transcriptional activation of type I IFN and the activation of NF-κB, was previously observed in the context of different herpesvirus infections [16–20, 39, 40]. By focusing on interactions that involve endogenously expressed host proteins, our findings provide new insight on the cellular targets of the viral enzymes and suggest possible new strategies for interfering with infection.

Using an unbiased approach based on co-immunoprecipitation and mass spectrometry, we have identified several members of the 14-3-3-family of molecular scaffold proteins as interacting partners of the EBV encoded BPLF1 (Fig 1). It should be noted that the N-terminal catalytic domain of BPLF1 was used as bait in these experiments. While many additional interactions are likely to occur with the much larger intact protein, the relevance of the isolated N-terminal domain is supported by the finding that the corresponding fragment is generated from endogenously expressed BPLF1 by caspase-1-dependent cleavage [21].

The 14-3-3 proteins are a family of conserved regulatory molecules expressed in all eukaryotic cells that bind as homo- or heterodimers to a multitude of functionally diverse proteins, including kinases, phosphatases, and transmembrane receptors [33, 41]. More than 300 signaling proteins have been reported as 14-3-3 ligands [42]. Thus, through binding to 14-3-3, BPLF1 could participate in the regulation of many signaling pathways. By focusing on the shared interaction networks, we have identified two cellular ubiquitin ligases, CUL1 and TRIM25, as possible partners in this regulation (Fig 1C). We have previously shown that BPLF1 binds to and promotes the functional inactivation of Cullin-RING-Ligases (CRLs) by deNeddylating the cullin scaffold, which induces cell cycle arrest and promotes virus replication [10, 43]. Different 14-3-3 family members participate in the regulation of the cell cycle by controlling the stability and nuclear transport of several CRL substrates [44, 45], and are therefore likely to be members of the signaling complexes regulated by BPLF1 in the nucleus. In the cytoplasm, 14-3-3 and TRIM25 act as essential co-factors in the signaling of viral nucleic acid sensors such as RIG-I and MDA5 [28, 46]. 14-3-3 stabilizes the interaction of TRIM25 with RIG-I, facilitating thereby the ubiquitination of RIG-I [28], and the translocation of the activated complexes to Mitochondrial Anti-Viral Signaling proteins (MAVS) for downstream signaling [29]. It is noteworthy that later steps in the pathway leading to activation of the NF-kB and IRF3 transcription factors are also regulated by ubiquitination [47]. However, the finding that the BPLF1 mediated inhibition of IRF3 nuclear translocation and transcription of IFN and IFN-regulated genes (Figs 2 and 3) is associated with the formation of a tri-molecular complex with 14-3-3 and TRIM25, points to the RIG-I signalosome as the primary target of the viral deconjugase.

Our findings suggest several possible mechanisms by which BPLF1 could interfere with the activity of the signalosome. We have shown that both catalytically active and inactive BPLF1 form a stable tri-molecular complex with 14-3-3 and TRIM25, independently of activation of the signaling pathway by treatment with poly(I:C) or expression of the constitutively active RIG-I-2CARD (Fig 4). RIG-I was not detected in the complex, which is in line with the finding that a conformational change induced by Pathogen-Associated Molecular Patterns (PAMPs) is required for binding to TRIM25 [48]. A fast migrating 14-3-3 species was found to be strongly enriched in the complexes (Fig 4C). Due to the low recovery, very similar size of the isoforms and poor performance of the specific antibodies, we could not determine whether this corresponds to a distinct 14-3-3 splice variant or whether it may be due to changes in the levels of posttranslational modifications, such as phosphorylation that was shown to have profound effects on the binding capacity of 14-3-3.

We have shown that the TRIM25 found in tri-molecular complexes with 14-3-3 and the catalytically active BPLF1 is auto-ubiquitinated (Figs 4 and 5). It is noteworthy that ubiquitinated TRIM25 was hardly detected in total cell lysates but was robustly enriched in the BPLF1 immunoprecipitates, suggesting that binding to the viral protein is required for this effect. Structural studies have shown that in order to act as a ligase TRIM25 must form dimers and oligomers, which allows correct position of the substrate and Ub-loaded E2 [37, 49]. This requirement was confirmed in our experiments by the finding that ubiquitinated species are preferentially pulled-down in cross-immunoprecipitation assays from lysates of cells expressing differently tagged TRIM25 (Fig 5C). Active BPLF1 may promote TRIM25 oligomerization in different ways. Stabilization of the protein via inhibition of ubiquitin dependent proteolysis may play an important role. The linear ubiquitin ligase assembly complex (LUBAC) was shown to inhibit IFN-responses by targeting TRIM25 for proteasomal degradation [36], which is inhibited by the cellular ubiquitin deconjugase USP15 [50]. Thus, by mimicking the activity of USP15, BPLF1 may favor the assembly of active complexes. The concomitant recruitment of 14-3-3 may also contribute by stabilizing the TRIM25 oligomers. It is noteworthy that TRIM25 was shown to serve as a specific ligase for 14-3-3σ and promote its proteasomal degradation in breast cancer cells [27]. In addition, TRIM25 was shown to mediate ISG15 modification of 14-3-3σ [51], which may alter the binding and chaperone properties of 14-3-3 by interfering with the formation of active homo- and heterodimers. Conceivably, via the formation of a tri-molecular complex with 14-3-3 and TRIM25, active BPLF1 could interfere with these Ub and UbL conjugation events and regulate both the stability and function of its binding partners. While serving as a clear sign of oligomer formation and ligase activation, the functional significance of TRIM25 autoubiquitination remains uncertain. Interestingly, a TRIM25 mutant lacking the major ubiquitination site on Lys117 was shown to be functionally similar to wild type TRIM25 with respect to its ability to ubiquitinate RIG-I-2CARD and induce RIG-I-mediated activation of type-I IFN [36].

In sharp contrast with the findings that BLPF1 does not impair and may even enhance the activity of TRIM25, we found that expression of the viral enzyme is associated with failure to detect K63-linked ubiquitination of the endogenous RIG-I when the pathway is activated either by poly(I:C) treatment or by expression of the constitutively active 2CARD construct. This was not due to inability to recruit RIG-I to the active ligase since, in spite of lower levels of expression in cells expressing active BPLF1, a comparable fraction of RIG-I was recovered in the TRIM25 immunoprecipitates (Fig 6A). Conceivably, association of the viral deconjugase with the active TRIM25 ligase may counteract ubiquitination and promote the release of RIG-I from the complex. Alternatively, ubiquitinated RIG-I might be released from the complex, possibly due to weaker interaction with auto-ubiquitinated TRIM25. The release of ubiquitinated RIG-I would interrupt the signaling cascade since the formation of a tri-molecular complex with TRIM25 and 14-3-3 is required for translocation to MAVS [28]. Free ubiquitinated RIG-I could be targeted by cellular deconjugase or even by BPLF1 that is not bound in the TRIM25:14-3-3 complex. It is important to stress that, while deubiquitination of RIG-I and other members of the signaling cascade was previously reported for several BPLF1 homologs encoded by human herpesviruses [16, 17, 39, 40], we have found that all the homologs tested share the capacity to bind to 14-3-3 and TRIM25, and promote TRIM25 autoubiquitination (Fig 7), suggesting that this early step of the signaling cascade is a common target of the viral enzymes.

Collectively, our findings illustrate a previously unrecognized mechanism by which herpesviruses may interfere with the activity of Ub and UbL regulated signaling networks to counteract the activation of innate anti-viral responses. The large tegument proteins involved in this escape strategy are expressed during the productive virus cycle, suggesting that their capacity to halt cellular defenses may contribute to extend the time window under which effective virus replication may occur. In addition, the proteins are also packaged into infectious virus particles and their delivery to newly infected cells could participate in the cellular reprogramming that allows the successful establishment of infection. While outside of the scope of this study, a precise characterization of the mechanism by which the viral enzymes impair the activity of the TRIM25 ligase, and mapping the sites of interaction with the TRIM25:14-3-3 complex will be important steps toward the identification of new potential targets for antiviral treatment.

## Materials and methods

### Chemicals

DL-Dithiothreitol (DTT, D0632), N-Ethylmaleimide (NEM, E1271), Iodoacetamide (I1149), IGEPAL CA-630 (NP40, I3021), Triton X-100 (T9284), bovine serum albumin (BSA, A7906), Sodium dodecyl sulfate (SDS, L3771), Tween-20 (P9416), Ethylenediaminetetraacetic acid disodium salt dehydrate (EDTA, E4884) and Trizma base (Tris, 93349), Sodium butyrate (NaBu, B5887) and 12-O-tetradecanoylphorbol-13-acetate (TPA, 4174) were purchased from Sigma-Aldrich (St. Louis, MO, USA). Complete protease inhibitor cocktail (04693116001), phosphatase inhibitor cocktail (04906837001) and alkaline phosphatase (11097075001) were purchased from Roche Diagnostic (Mannheim, Germany). Ciprofloxacin (17850) was purchased from Fluka (Buchs, Switzerland). Poly(I:C) (LMW)/LyoVec (tlrl-picwlv) was purchased from Invivogen (Toulouse, France).

### Antibodies

Antibodies and their manufacturers were: mouse anti-β-actin clone AC-15 (1:5000, A5441), mouse anti-FLAG clone M2 (1:4000, IF: 1:500; F1804) and mouse anti-polyhistidine clone HIS-1 (1:2000, H1029) from Sigma-Aldrich; goat anti-FLAG (IF 1:500; ab1257) from Abcam; mouse anti-HA clone 12CA5 (1:2000; 11583816001) from Roche; mouse anti-RIG-I clone 1C3 (1:1000; MABF297) from EMD-Millipore (Darmstadt, Germany); mouse anti-pan14-3-3 clone H-8 (1:1000; sc-1657), rabbit anti-14-3-3β clone C20 (1:1000; sc-628), rabbit anti-14-3-3γ clone C-16 (1:1000; sc-731), rabbit anti-14-3-3θ clone C-17 (1:1000; sc-732), rabbit anti-14-3-3ζ clone C-16 (1:1000; sc-1019), rabbit anti-14-3-3ε clone T-16 (1:1000; sc-1020) from Santa-Cruz Biotechnology (Santa Cruz, CA, USA); rabbit anti-14-3-3η clone D23B7 (1:1000; #5521), rabbit anti-MDA5 clone D74E4 (1:1000; #5321), rabbit anti-RIG-I clone D14G6 (1:1000; #3743), rabbit anti-IRF-3 clone D6I4C (1:1000, IF 1:400; #11904), rabbit anti-pIRF-3 clone 4D4G (1:1000; #4947) and mouse anti-GST clone 26H1 (1:1000, IF: 1:800; #2624) from Cell-Signaling Technologies (Danvers, MA, USA); rabbit anti TRIM25 clone EPR7315 (1:2000; ab167154) from Abcam (Cambridge, MA, USA); mouse anti-V5 clone 2F11F7 (1:2000; 37–7500) from Invitrogen (Rockford, IL, USA); mouse anti-HA.11 clone 16B12 (1:1000; 901501) from BioLegend (San Diego, CA, USA). Alexa Fluor 488, 555 and 647 conjugated secondary antibodies were from Thermo Fisher (A21206, A31570 and A21447, respectively).

### Plasmids

Plasmids encoding 3xFLAG-BPLF1 (amino acid residues 1–235 and amino acid residues 1–325), the catalytic mutant BPLF1 C61A and 3xFLAG-KSHV-ORF64 (amino acid residues 1–205), and HA-tagged ubiquitin were described previously [10, 43]. His_6_-tagged pcDNA3-ISG15 was kindly provided by Bret A. Hassel (University of Maryland). 3xFLAG-tagged HSV-UL36 (amino acid residues 1–293) was kindly provided by Lars Dolken, University of Wurzberg, Germany and 3xFLAG-HCMV-UL48 (amino acid residues 1-263) was kindly provided by Luka Cicin-Sain, Helmholtz Center for Infection Research. Plasmid pRK5-HA-UbK63 that encode for ubiquitin polypeptides where all Lys residues are mutated to Arg except for Lys63 were kindly provided by Harald Wodrich, University of Bordeaux. Plasmids encoding for GST-2CARD, V5-TRIM25 and V5-TRIM25ΔRING were kindly provided by Jae Jung, University of Southern California. pFLAGCMV2-TRIM25 (Addgene plasmid # 12449) and pcDNA3.0-HA-TRIM25 (Addgene plasmid # 12452) were gifts from Dong-Er Zhang [51]. The BZLF1 expression plasmid was a generous gift from Regina Feederle, Helmholz Zentrum, Munich.

### Construction of BPLF1 catalytic mutant recombinant EBV

The recombinant EBV wild-type B95-8 strain (rB95-8/p2089) was constructed by introducing a prokaryotic F-plasmid, which carries the chloramphenicol (cam) resistance gene, the gene for the green fluorescent protein (GFP), and the hygromycin resistance gene, into the EBV strain B95-8 [52]. We performed En passant mutagenesis [53] to construct the BPLF1 catalytic mutant (B769) in rB95-8 where amino acid 61 of BPLF1 was replaced from Cys to Ala (C61A, nucleotide 71346:71347 (V01555.2) DNA changed from CA into GC) [10] in the bacterial strain GS1783. The linear targeting DNA fragment used for En passant mutagenesis carried the kanamycin resistance gene flanked by sequences homologous to the BPLF1 including the C61A mutation that was amplified from the pEP-Kans plasmid using the primers 1108 (CTCTTGACCA GGTAGAGGAC GCAGTTGCTG ACGGCCTGGA TGCCGGCAAA GCGGCCAAAC TAGGGATAAC AGGGTAATCG ATTT) and 1109 (GTGCAACCAG GCCCACTGCA AGTTTGGCCG CTTTGCCGGC ATCCAGGCCG TCAGCAACTG CGCCAGTGTT ACAACCAATT AACC). The integrity of mutant correct clone was confirmed by restriction enzyme digestion and sequencing of the mutated regions.

### Cell lines and transfection

HeLa cells (ATCC RR-B51S), 293T cells (ATCC CRL3216) and U2OS (ATCC HTB-96) cells were cultured in Dulbecco’s minimal essential medium (DMEM, Sigma-Aldrich), supplemented with 10% FCS (Gibco-Invitrogen), ciprofloxacin (10 μg/ml) and maintained in a 37°C incubator in 5% CO_2_. The EBV negative gastric carcinoma line AGS [54] and the EBV converted AGS-Bx1 cell line (kindly provided by Alan Chiang, Hong Kong University, Hong Kong) [55] were cultured in Dulbecco's Modified Eagle Medium: Nutrient Mixture F-12 (DMEM/F12) (GIBCO-Invitrogen, Carlsbad, USA) supplemented with 10% Fetal Bovine Serum. AGS-Bx1 was cultured in complete medium supplemented with 500 μg/ml Geneticin (GIBCO-Invitrogen). HEK293 cells infected with BPLF1 wild type or mutant EBV were cultured in DMEM supplemented with 10% FCS (Gibco-Invitrogen) 10 μg/ml ciprofloxacin and 100 μg/ml Hygromycin B (Calbiochem) to select for EBV carrying cells.

### Induction of the productive virus cycle

To induce the productive virus cycle AGS-Bx1 cells were cultured in medium supplemented with 30 ng/ml TPA and 0.5 mM NaBu for 48 h and HEK293-EBV cells were transfected with a plasmid expressing the EBV BZLF1 transactivator. Plasmid transfection was performed using the jetPEI kit (Polyplus transfection; Illkirch, France) or Lipofectamine 3000 (Life technologies, US) as recommended by the manufacturer. The efficiency of induction was monitored by probing western blots with antibodies to the viral proteins BZLF1 and BMRF1. Expression of the BPLF1 mRNA was measured by qRT-PCR.

### Tandem mass spectrometry and bioinformatics analysis

Ten 10 cm dishes of HeLa cells were transfected with ~20 μg plasmids encoding FLAG-BPLF1 (amino acid residues 1–325), FLAG-BPLF1C61A or FLAG-empty vector. Two days later, the cells were lysed in NP-40 lysis buffer (50 mM Tris-HCl pH 7.6, 150 mM NaCl, 5mM MgCl2, 1 mM EDTA, 1mM DTT, 1% Igepal, 10% glycerol) supplemented with protease inhibitor cocktail, 20 mM NEM and 20 mM Iodoacetamide. Ten mg of clarified lysates were mixed with 100 μl of a 50% slurry of anti-FLAG-conjugated agarose gel (A-2220; Sigma) and incubated for 4h at 4°C. After five times washing with NP-40 lysis buffer, bound proteins were eluted with 60 μl of FLAG peptide (300 μg/ml, F-4799; Sigma) and separated by NuPAGE 4–12% Bis-Tris gradient gel electrophoresis (Life Technologies). Silver staining was performed with the SilverQuest Silver Staining Kit (Thermo Scientific) and each lane was excised into 1 cm^2^ pieces using a sterile scalpel. The gel pieces were trypsinized and analyzed by Nano liquid chromatography tandem mass spectrometry (LC/MS/MS) at the Proteomics Mass Spectrometry facility of the SciLifeLab, Stockholm. Only proteins that were absent in duplicate samples of the FLAG-empty vector control and that were detected by at least 2 unique spectral counts in either or both the FLAG-BPLF1 and FLAG-BPLF1C61A immunoprecipitates were considered as positive hits. Functional interaction network analysis was performed using the Search Tool for the Retrieval of Interacting Genes (STRING) database v. 9.0 [56]. STRING integrates information on physical interactions and functional relationships identified by high-throughput biochemical analysis, mining of databases and literature, and prediction from genomic context analysis, into Protein-Protein-Interaction (PPI) networks. Proteins showing at least one interaction in the human PPI network with a threshold score >0.8 were visualized using Cytoscape v. 3.0.2 [57]. The functional annotation clustering tool of the DAVID bioinformatics resource [58] was used to identify the overrepresentation of genes in particular functional categories and pathways databases including Gene Ontology [59], Panther [60] and the Kyoto Encyclopedia of Genes and Genomes (KEGG) [61]. Protein interaction networks were visualized using the CIRCOS software [62]. The 14-3-3Pred [63] and Scansite Motifscan [64] softwares were used to predict putative 14-3-3 binding site in BPLF1.

### Immunoblotting and immunoprecipitation

For immunoblotting and co-immunoprecipitation cells harvested 48hrs post transfection were lysed in NP-40 lysis buffer (50 mM Tris-HCl pH 7.6, 150 mM NaCl, 5mM MgCl2, 1 mM EDTA, 1mM DTT, 1% Igepal, 10% glycerol) supplemented with protease inhibitor cocktail, 20 mM NEM and 20mM Iodoacetamide. Protein concentration was measured with a protein assay kit (Bio-Rad Laboratories). For co-immunoprecipitation, the cell lysates were incubated for 4 hrs or overnight with anti-FLAG agarose affinity gel (A-2220; Sigma) or Monoclonal Anti-HA-Agarose, Clone HA-7 (A2095; Sigma), followed by washing with lysis buffer and elution with 3x-FLAG peptide (F4799; Sigma) or HA peptide (I2149; Sigma) respectively at a concentration of 300 μg/ml. For co-immunoprecipitation of endogenous TRIM25 and RIG-I the cell lysates were incubated for 4 hrs with specific antibodies followed by 2 hrs with protein-G coupled Sepharose beads (17-0885-01; GE Healthcare). Immunocomplexes were washed with lysis buffer and elution was performed by boiling for 10 mins in 2x SDS-PAGE loading buffer. GST pull down was performed with glutathione-Sepharose 4B beads (17-0756-01; GE Healthcare). To resolve protein complexes, cell pellets were lysed in 100 μl NP-40 lysis buffer supplemented with 1% sodium dodecyl sulfate (SDS). Before immunoprecipitation NP-40 buffer was added to reach a final concentration of 0.1% SDS. Immunocomplexes were washed with lysis buffer containing 0.1% SDS. Equal amounts of proteins were fractionated in a polyacrylamide Bis-Tris 4–12% gradient precast gels (Invitrogen). After transfer to poly-vinylidene difluoride (PVDF) membranes (Millipore), the blots were blocked in Tris-buffered saline containing 5% non-fat milk and 0.1% Tween 20 and incubated with primary antibodies either for 1h at room temperature or overnight at 4°C followed by incubation for 1 hr with the appropriate horseradish peroxidase-conjugated secondary antibodies. The complexes were visualized by chemiluminescence (ECL; GE Healthcare).

### Quantitative RT-PCR

Messenger RNA expression of components of the RIG-I signaling pathway was measured by qRT-PCR. The sequences of the qPCR primers are listed in Supporting information, S1 Table. Briefly, total RNA was isolated using the RNeasy Mini Kit (Qiagen, Hilden, Germany) and reverse transcribed using a high capacity reverse transcription kit (Applied Biosystems, CA, USA) with condition: 25°C for 10 mins followed by 37°C for 120 mins and 85°C for 5 mins. Quantitative RT-PCR assays were setup using the Power SYBR Green PCR Master Mix (Applied Biosystems by Life Technologies, Woolston Warrington, UK) using 100 nM of primer pairs with cycling conditions: initial 50°C 2 mins, denaturation 95°C 10 mins, followed by 40 cycles of 95°C for 15 secs, 60°C for 1 min. Melting curves were run by incubating the reaction mixtures at 95°C for 15 secs, 60°C for 20 secs, 95°C for 15 secs, ramping from 60°C to 95°C at 1°C/sec. The values were normalized to an endogenous GAPDH. Fold change was calculated as: Fold Change = 2-Δ(ΔCt) where ΔCt = Ct target−Ct housekeeping and Δ(ΔCT) = ΔCt treated− ΔCt untreated, according to the Minimum Information for Publication of Quantitative Real-Time PCR Experiments (MIQE) guidelines.

### Immunofluorescence and confocal microscopy

HeLa cells were grown to semi-confluence in Dulbecco’s minimal essential medium containing 10% fetal calf serum and 10 μg/ml ciprofloxacin on glass cover slips and transfected with the indicated plasmids using the jetPEI kit as recommended by the manufacturer. After 24 hrs the cells were fixed in 4% paraformaldehyde (Merck, 100496) and permeabilized using 0.1% Triton X-100 in PBS, followed by blocking with 0.12% glycine (Fisher Scientific, G46-1) in PBS for 10 mins, and 3% bovine serum albumin (BSA, Sigma, A7906) in PBS for 15 mins at room temperature. The cells were triple labeled in 3% BSA-PBS using rabbit anti-IRF3, mouse anti-GST and goat anti-FLAG antibodies followed by the appropriate Alexa Fluor 488, 555 or 647 conjugated secondary antibodies. The coverslips were mounted cell side down on object glasses with Mowiol (Calbiochem, 475904) containing 50 mg/ml 1,4-diazabicyclo[2.2.2]octane (Dabco; Sigma, D-2522) as anti-fading agent and 2 μg/ml Hoechst 33258 (Sigma, 861405) to stain the nuclei. The samples were imaged using a confocal scanning laser microscope (Zeiss LSM510 META) and 2.5 μm optical sections were acquired. Images were analyzed by scoring nuclei that were either negative or positive for endogenous IRF3 in cells expressing both GST and FLAG fluorescence or GST fluorescence alone.

## Supporting information

S1 Table Primer sequences used for qPCR. (PDF)

S1 Fig Bioinformatics analysis of BPLF1 mass spectrometry data. **A**. Silver-stained gel of proteins co-immunoprecipitated with FLAG-BPLF1 from HeLa cells. The BPLF1 is indicated by an arrow. Each lane of the gel was cut into equal sections and each section was divided into 1 cm^2^ pieces. The gels were trypsinized and analyzed by LC/MS/MS. **B**. Venn Diagram illustrating the overlap between proteins interacting with BPLF1 and BPLF1C61A identified by mass spectrometry. A total of 277 proteins were detected in FLAG-immunoprecipitates of cells expressing the BPLF1 proteins but not in the control FLAG-empty vector in two independent experiments. Of these, 72 bound to both the active and catalytic mutant BPLF1 while 187 bound exclusively to the active enzyme and 18 bound only to the mutant. **C**. Gene Ontology Biological Process enrichment analysis. Statistically significant (P-value <0.05) enriched terms in the GO biological process category are shown. BPLF1 interacting proteins are predicted to be involved in RNA metabolism, protein localization and transport, regulation of the cell cycle and DNA damage and immune responses. Several interacting proteins including E3 ligases and proteasome subunits are involved in ubiquitin-dependent processes. **D**. Functional network analysis. String interaction network showing experimentally validated interaction of the 277 BPLF1 interacting proteins. Among those, 116 proteins were found in a unique network where highly interacting nodes include proteasome subunits, EGFR, components of the RNA metabolism and nuclear export complex and the 14-3-3 family of scaffold proteins.
(TIF)

S2 Fig The interaction of BPLF1 with 14-3-3 is not dependent on phosphorylation. Total cell lysates were prepared in NP-40 lysis buffer containing protease inhibitors but devoid of EDTA and phosphatase inhibitor. One mg of total lysate was treated with 250 units of calf intestine phosphatase (Roche, 11 097 075 001) for 1 hr at 37°C followed by FLAG immunoprecipitation. Western blots were probed with the indicated antibodies. Treatment with phosphatase did not affect the efficiency of immunoprecipitation. One representative experiment out of 2 is shown.
(TIF)

S3 Fig BPLF1 does not affect the turnover of endogenous 14-3-3 proteins but may affect their ubiquitination. **A**. Western blots of cells expressing the indicated FLAG-tagged plasmids were probed with antibodies specific for the indicated 14-3-3 isoforms. One aliquot of the cells was treated with 10 μM of the proteasome inhibitor MG132 for 6 hrs before harvesting. Expression of catalytically active BPLF1 did not affect the steady state levels of the proteins. **B**. The effect of BPLF1 on the ubiquitination of 14-3-3 was investigated in cells overexpressing HA-Ub. HA-immunoprecipitates were probed with a pan-14-3-3 antibody. Slow migrating species of size corresponding to mono- and di-ubiquitinated 14-3-3 were detected in cells transfected with the FLAG-vector and catalytic mutant BPLF1 but not in cells expressing the active enzyme. A previously described longer version of the BPLF1 N-terminal domain that is processed in cells to yield a ≈ 235 amino acid species was used in the experiment.
(TIF)

S4 Fig Transfected TRIM25 is modified by ubiquitin but not by ISG15. **A**. TRIM25 from HeLa cells was immunoprecipitated from HeLa cells co-transfected with 6xHis-ISG15 and the indicated FLAG-tagged plasmids. Western blots were probed with antibodies to TRIM25 and the HIS tag. High molecular species TRIM25 were not detected by the HIS antibody indicating that BPLF1 does not promote TRIM25 ISGylation. **B**. HeLa cells co-transfected with the indicated plasmids were lysed in NP-40 buffer with or without addition of the cysteine protease inhibitors NEM and iodoacetamide. After incubation of 1 h at 37°C the lysates were fractionated by SDS-PAGE and western blots were probed with the anti-HA antibody. Omission of NEM and iodoacetamide was accompanied by disappearance of the high molecular weight species supporting the conclusion that overexpressed TRIM25 is ubiquitinated and the modification is increased in cells expressing catalytically active BPLF1. **C**. BPLF1 can hydrolyze both K48- and K63-linked polyubiquitin chains. HeLa cells co-transfected with the indicated FLAG-tagged plasmids and plasmids expressing HA-UbK48 or HA-UbK63. Western blots were probed with anti-HA antibodies.
(TIF)

S5 Fig Functional assay confirming the enzymatic activity of BPLF1 and the functional homologs encoded by other human herpesviruses. NP-40 lysates of cells expressing FLAG-tagged versions of the N-terminal domain of the indicated homologs were incubated for 1 hr at 37°C with 0.5 μg of the Ub-VME functional probe. After fractionation by SDS-PAGE and blotting on PVDF membranes the viral proteins were detected with an anti-FLAG antibody. Enzymatic activity is confirmed by the appearance of a slower migrating species of size corresponding to cross-linking of the Ub-VME probe.
(TIF)
