# Supplementary material for: Herpesvirus deconjugases inhibit the IFN response by promoting TRIM25 autoubiquitination and functional inactivation of the RIG-I signalosome
Source: PLoS Pathog. 2018 Jan 22;14(1):e1006852. doi: 10.1371/journal.ppat.1006852 (PMC5794190; doi:10.1371/journal.ppat.1006852)
Supplement: S1 Table — (PDF) [file ppat.1006852.s001.pdf]

Table ST1. Primers used for qPCR.

Name	Sequence: (5' to 3')
IFNβ Fwd	TCCAAATTGCTCTCCTGTTG
IFNβ Rev	GCAGTATTCAAGCCTCCCAT
RIG-I Fwd	ATCCCAGTGTATGAACAGCAG
RIG-I Rev	GCCTGTAACTCTATACCCATGTC
MDA5 Fwd	TGGTCTCGTCACCAATGAAA
MDA5 Rev	CTCCTGAACCACTGTGAGCA
GAPDH Fwd	TGGGCTACACTGAGCACCAG
GAPDH Rev	GGGTGTCGCTGTTGAAGTCA
BPLF1 Fwd	GCAGTCCCCGGACATGAAAT
BPLF1 Rev	TTAGCCTGGCCTGGGTCATT
