# Supplementary figures and images for: Herpesvirus deconjugases inhibit the IFN response by promoting TRIM25 autoubiquitination and functional inactivation of the RIG-I signalosome
Source: PLoS Pathog. 2018 Jan 22;14(1):e1006852. doi: 10.1371/journal.ppat.1006852 (PMC5794190; doi:10.1371/journal.ppat.1006852)

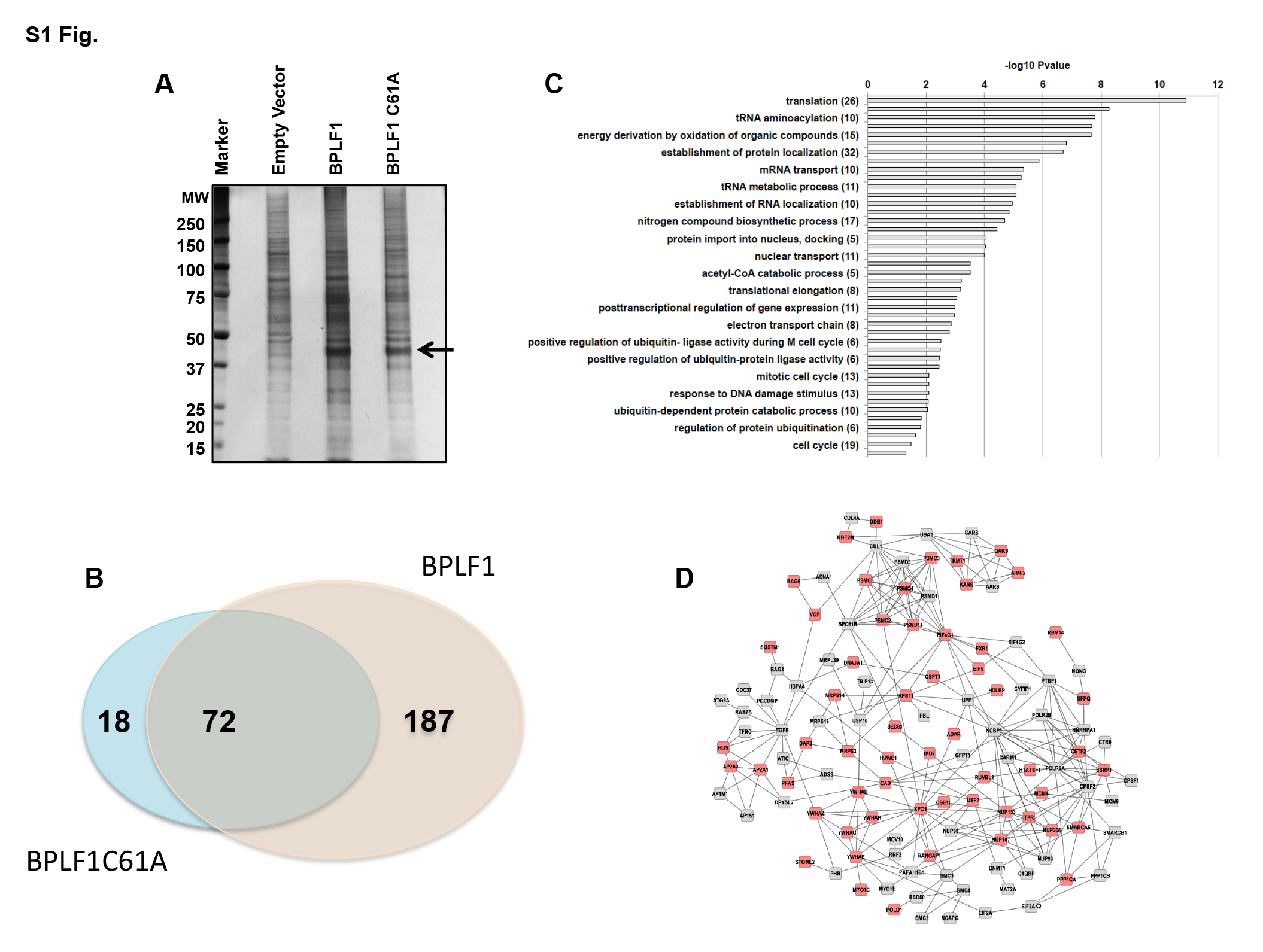

Supplement: S1 Fig — A. Silver-stained gel of proteins co-immunoprecipitated with FLAG-BPLF1 from HeLa cells. The BPLF1 is indicated by an arrow. Each lane of the gel was cut into equal sections and each section was divided into 1 cm2 pieces. The gels were trypsinized and analyzed by LC/MS/MS. B. Venn Diagram illustrating the overlap between proteins interacting with BPLF1 and BPLF1C61A identified by mass spectrometry. A total of 277 proteins were detected in FLAG-immunoprecipitates of cells expressing the BPLF1 proteins but not in the control FLAG-empty vector in two independent experiments. Of these, 72 bound to both the active and catalytic mutant BPLF1 while 187 bound exclusively to the active enzyme and 18 bound only to the mutant. C. Gene Ontology Biological Process enrichment analysis. Statistically significant (P-value <0.05) enriched terms in the GO biological process category are shown. BPLF1 interacting proteins are predicted to be involved in RNA metabolism, protein localization and transport, regulation of the cell cycle and DNA damage and immune responses. Several interacting proteins including E3 ligases and proteasome subunits are involved in ubiquitin-dependent processes. D. Functional network analysis. String interaction network showing experimentally validated interaction of the 277 BPLF1 interacting proteins. Among those, 116 proteins were found in a unique network where highly interacting nodes include proteasome subunits, EGFR, components of the RNA metabolism and nuclear export complex and the 14-3-3 family of scaffold proteins. (TIF) [file ppat.1006852.s002.tif]

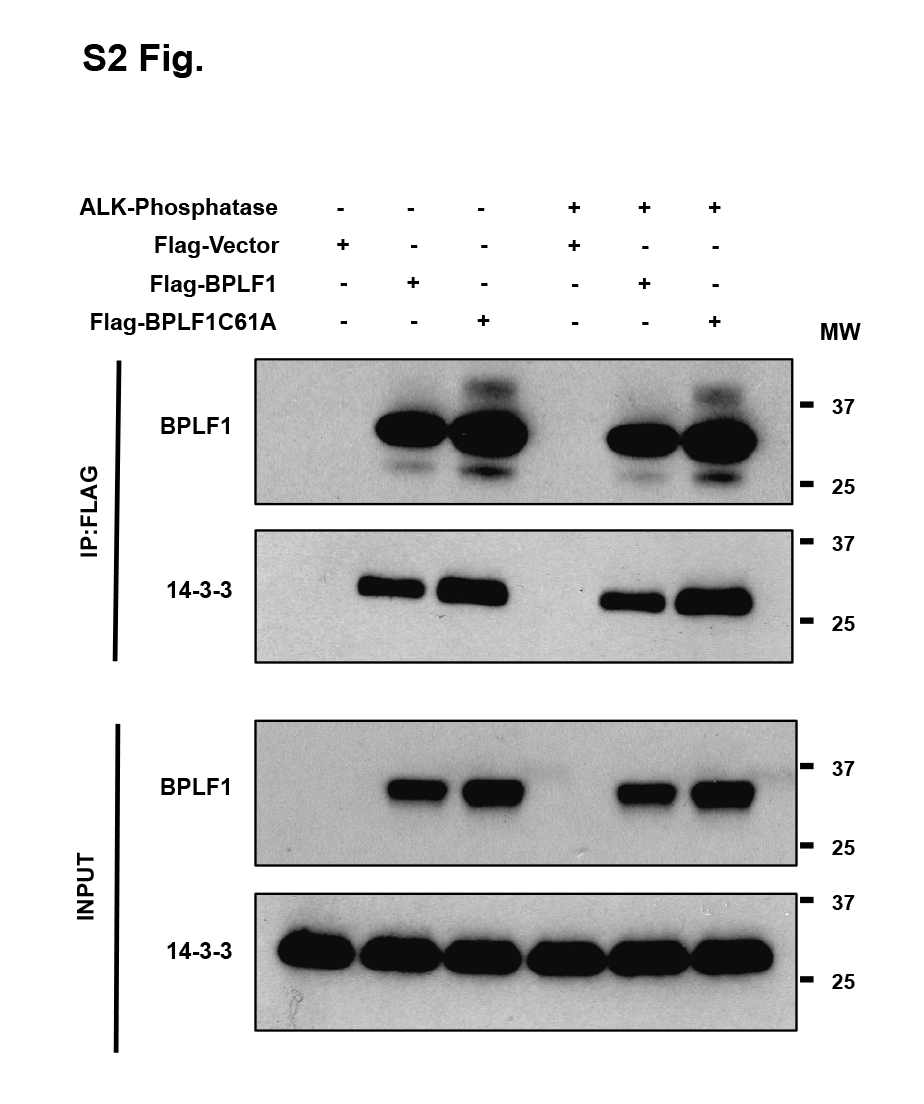

Supplement: S2 Fig — Total cell lysates were prepared in NP-40 lysis buffer containing protease inhibitors but devoid of EDTA and phosphatase inhibitor. One mg of total lysate was treated with 250 units of calf intestine phosphatase (Roche, 11 097 075 001) for 1 hr at 37°C followed by FLAG immunoprecipitation. Western blots were probed with the indicated antibodies. Treatment with phosphatase did not affect the efficiency of immunoprecipitation. One representative experiment out of 2 is shown. (TIF) [file ppat.1006852.s003.tif]

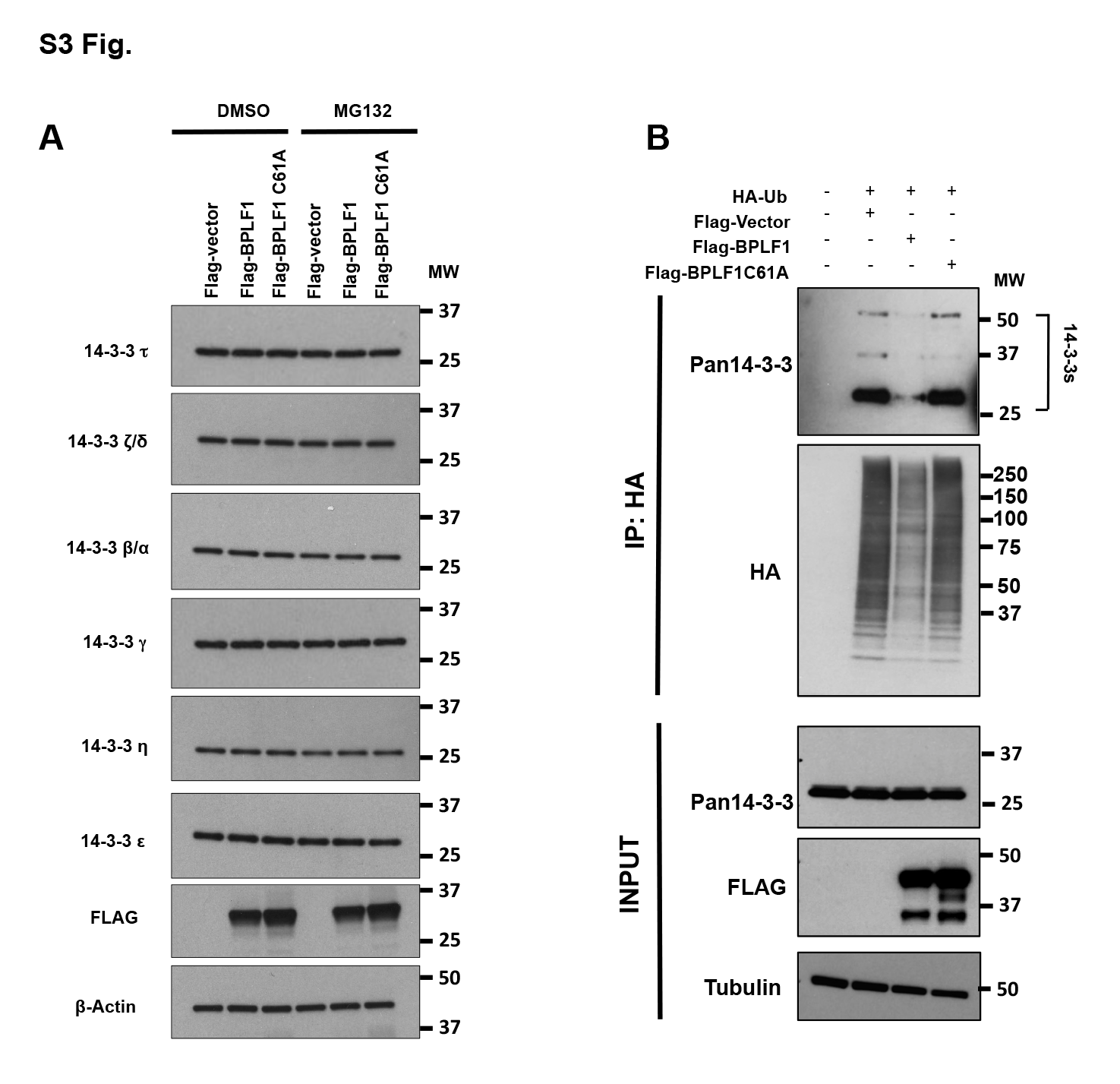

Supplement: S3 Fig — A. Western blots of cells expressing the indicated FLAG-tagged plasmids were probed with antibodies specific for the indicated 14-3-3 isoforms. One aliquot of the cells was treated with 10 μM of the proteasome inhibitor MG132 for 6 hrs before harvesting. Expression of catalytically active BPLF1 did not affect the steady state levels of the proteins. B. The effect of BPLF1 on the ubiquitination of 14-3-3 was investigated in cells overexpressing HA-Ub. HA-immunoprecipitates were probed with a pan-14-3-3 antibody. Slow migrating species of size corresponding to mono- and di-ubiquitinated 14-3-3 were detected in cells transfected with the FLAG-vector and catalytic mutant BPLF1 but not in cells expressing the active enzyme. A previously described longer version of the BPLF1 N-terminal domain that is processed in cells to yield a ≈ 235 amino acid species was used in the experiment. (TIF) [file ppat.1006852.s004.tif]

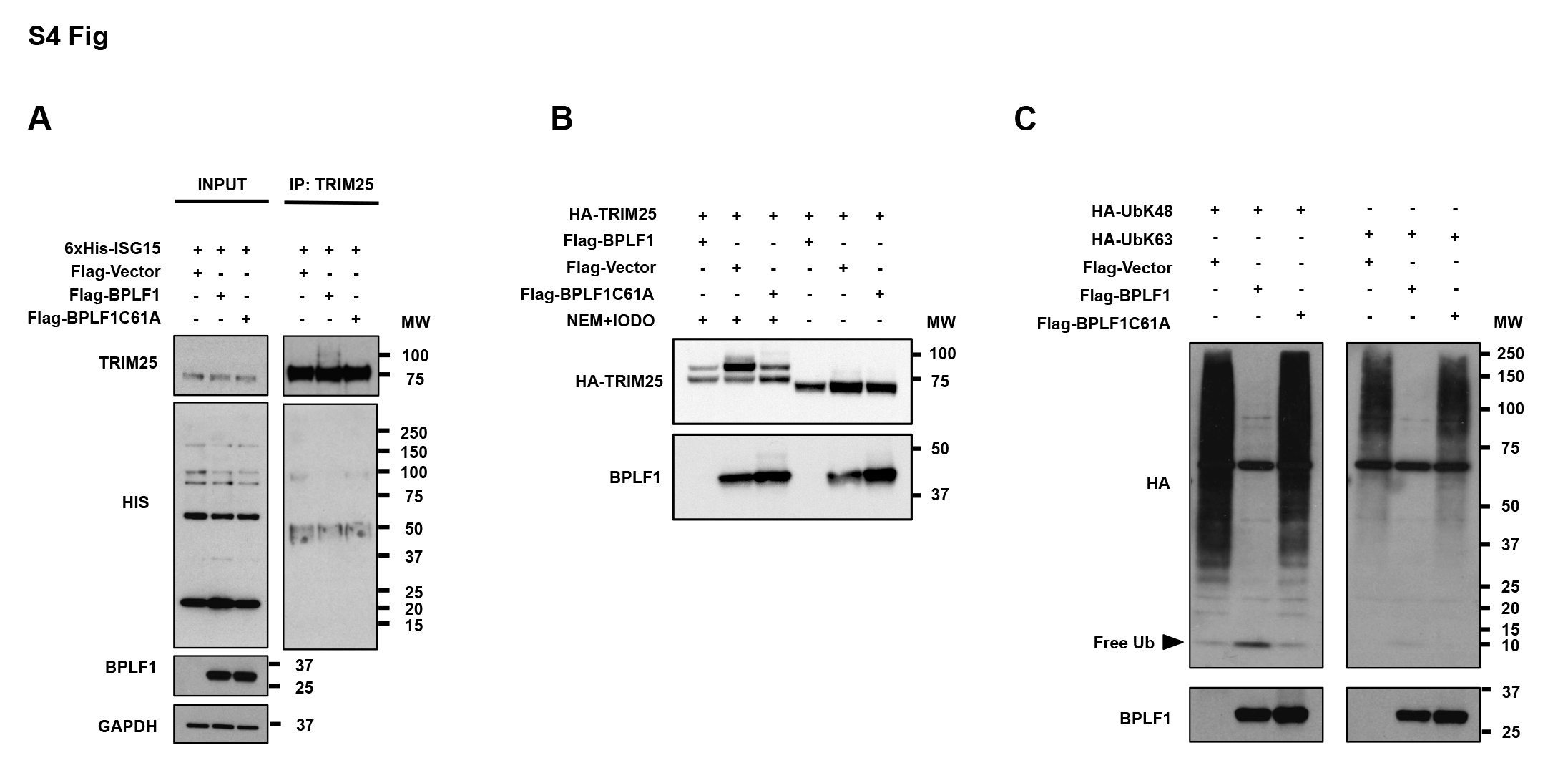

Supplement: S4 Fig — A. TRIM25 from HeLa cells was immunoprecipitated from HeLa cells co-transfected with 6xHis-ISG15 and the indicated FLAG-tagged plasmids. Western blots were probed with antibodies to TRIM25 and the HIS tag. High molecular species TRIM25 were not detected by the HIS antibody indicating that BPLF1 does not promote TRIM25 ISGylation. B. HeLa cells co-transfected with the indicated plasmids were lysed in NP-40 buffer with or without addition of the cysteine protease inhibitors NEM and iodoacetamide. After incubation of 1 h at 37°C the lysates were fractionated by SDS-PAGE and western blots were probed with the anti-HA antibody. Omission of NEM and iodoacetamide was accompanied by disappearance of the high molecular weight species supporting the conclusion that overexpressed TRIM25 is ubiquitinated and the modification is increased in cells expressing catalytically active BPLF1. C. BPLF1 can hydrolyze both K48- and K63-linked polyubiquitin chains. HeLa cells co-transfected with the indicated FLAG-tagged plasmids and plasmids expressing HA-UbK48 or HA-UbK63. Western blots were probed with anti-HA antibodies. (TIF) [file ppat.1006852.s005.tif]

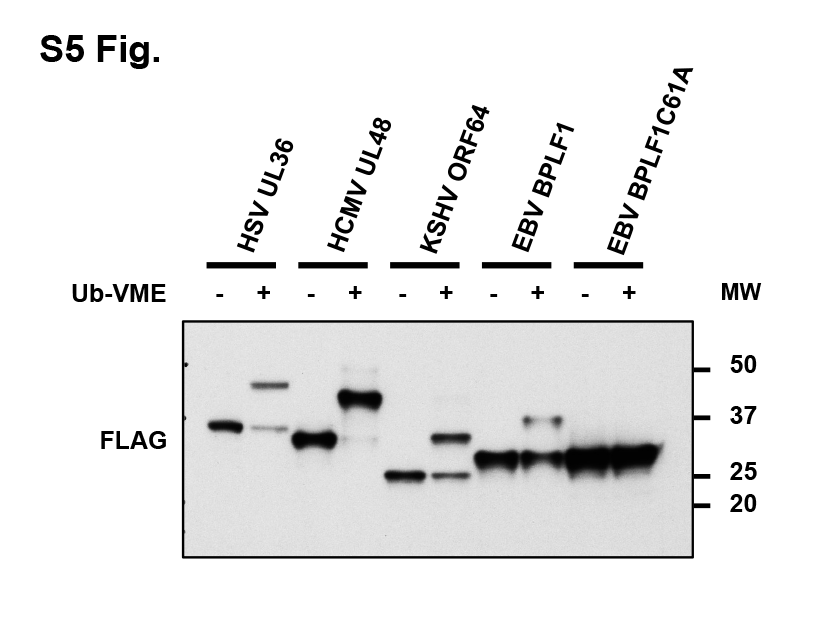

Supplement: S5 Fig — NP-40 lysates of cells expressing FLAG-tagged versions of the N-terminal domain of the indicated homologs were incubated for 1 hr at 37°C with 0.5 μg of the Ub-VME functional probe. After fractionation by SDS-PAGE and blotting on PVDF membranes the viral proteins were detected with an anti-FLAG antibody. Enzymatic activity is confirmed by the appearance of a slower migrating species of size corresponding to cross-linking of the Ub-VME probe. (TIF) [file ppat.1006852.s006.tif]
